# Anthocyanin concentration depends on the counterbalance between its synthesis and degradation in plum fruit at high temperature

**DOI:** 10.1038/s41598-017-07896-0

**Published:** 2017-08-09

**Authors:** Junping Niu, Guojing Zhang, Wenting Zhang, Vasilij Goltsev, Shan Sun, Jinzheng Wang, Pengmin Li, Fengwang Ma

**Affiliations:** 1 State Key Laboratory of Crop Stress Biology for Arid Areas, College of Horticulture, Northwest A&F University, Yangling, Shaanxi 712100 China; 2 Department of Biophysics and Radiobiology, Faculty of Biology, St. Kliment Ohridski University of Sofia, 8 Dr. Tzankov Blvd, 1164 Sofia, Bulgaria; 3 Shandong Institute of Pomology, Taian, Shandong 271000 China

## Abstract

Anthocyanin synthesis and degradation processes were analyzed at transcript, enzyme, and metabolite levels to clarify the effects of high temperature on the concentration of anthocyanin in plum fruit (*Prunus salicina* Lindl.). The transcript levels of *PsPAL*, *PsCHS*, and *PsDFR* decreased while those of *PsANS* and *PsUFGT* were similar at 35 °C compared with 20 °C. The activities of the enzymes encoded by these genes were all increased in fruits at 35 °C. The concentrations of anthocyanins were higher at 35 °C on day 5 but then decreased to lower values on day 9 compared with that at 20 °C. Furthermore, high temperature (35 °C) increased the concentration of hydrogen peroxide and the activity of class III peroxidase in the fruit. The concentration of procatechuic acid, a product of the reaction between anthocyanin and hydrogen peroxide, hardly changed at 20 °C but was significantly increased at 35 °C on day 9, indicating that anthocyanin was degraded by hydrogen peroxide, which was catalyzed by class III peroxidase. Based on mathematical modeling, it was estimated that more than 60–70% was enzymatically degraded on day 9 when the temperature increased from 20 °C to 35 °C. We conclude that at the high temperature, the anthocyanin content in plum fruit depend on the counterbalance between its synthesis and degradation.

## Introduction

Anthocyanins are a group of water-soluble pigments that accumulate in the cell vacuoles of plants. They are responsible for the red, purple, and blue colors in roots, leaves, flowers, fruit, and other plant tissues. Anthocyanins play a vital role in plant survival as attractants of pollinators/dispersers, repellents of herbivores, and light attenuators to protect cells against damage caused by intense light by absorbing yellow-green and ultraviolet light under various stress conditions^1–4^. Anthocyanins have also been shown to act as an antioxidant to scavenge reactive oxygen species (ROS) in plants suffering from oxidative stresses^5–8^.

To date, the biosynthetic pathway underlying anthocyanin production is well known. In most plant species, anthocyanins are synthesized from L–phenylalanine via the phenylpropanoid pathway catalyzed by phenylalanine ammonia lyase (PAL), chalcone synthase (CHS), chalcone-flavanone isomerase (CHI), flavanone-3-hydroxylase (F3H), dihydroflavonol 4-reductase (DFR), anthocyanidin synthase (ANS), and UDP-glycose:flavonoid 3-O-glycosyltransferase (UFGT), among other enzymes^9–11^. However, in contrast to the extensive knowledge regarding anthocyanin synthesis, little has been known about the anthocyanin degradation process in living plant tissues until a breakthrough was reported by Zipor *et al*.^8^. They isolated a vacuolar class III peroxidase (Prx) from the *Brunfelsia calycina* flower and demonstrated that Prx is responsible for the *in planta* degradation of anthocyanins^8^. This is the first demonstration of an enzyme that catalyzes anthocyanin degradation *in planta*, although in addition to Prx, other enzymes such as polyphenol oxidase and β–glucosidase have been suggested to be involved in anthocyanin degradation in fruit juices^12^.

Because both the synthesis and degradation of anthocyanins are enzymatically controlled *in planta*, changes in these two processes should benefit plants. For instance, the sun-exposed sides of apple and pear fruits often suffer from severe light stress during development. As a consequence, they possess a higher anthocyanin synthesis capacity and elevated anthocyanin concentrations compared with the shaded sides of the fruits^4, 13–15^. Anthocyanin usually accumulates to a greater extent in young or senescing leaves in contrast to healthy mature leaves^16, 17^. The young and senescing leaves are more sensitive to intense light mainly due to their much lower photosynthetic capacity. Therefore, the anthocyanins that are synthesized in the adaxial surface of the leaf could attenuate the light irradiated to the chloroplast and thus protect the leaves against photoinhibition/photodamage. Anthocyanins in the adaxial leaf tissues provide greater photoprotection than those in abaxial tissues but also predispose the tissues to increased shade acclimation and, consequently, reduced photosynthetic capacity. Thus, in the leaves of some understory plants of temperate and tropical forests, anthocyanin is synthesized in the abaxial side, which may represent a compromise based on costs/benefits^18^. In general, the relationship between anthocyanin synthesis and its function under different light conditions has been well explained in those studies.

Regarding the relationship between anthocyanin synthesis and temperature, it is very easy to understand why a low temperature often increases the concentration of anthocyanin, for example, in the leaves of evergreen species during the winter^19^. Anthocyanin may protect plants against various stresses. Similarly, the synthesis of anthocyanin is expected to be enhanced in response to high temperature stress, which may increase the production of ROS in mitochondria through respiration^20, 21^ and the cell membrane fluidity^22^, potentially facilitating the diffusion of ROS from mitochondria to other organelles. Indeed, anthocyanin may protect pear peel against high temperature damage during the cross stresses of high temperature coupled with intense light^23^. However, the concentration of anthocyanin in pear peel was also found to decrease in response to a high temperature^23^. In addition, Rowan *et al*.^24^ reported that the concentration of anthocyanin was reduced under low light coupled with high temperature conditions in red *Arabidopsis thaliana* plants over-expressing the PAP1 transcription factor. They suggested that the loss of anthocyanin was not related to catabolic enzymes but to a decline in the expression of biosynthetic genes and a continuous and relatively constant rate of non-enzymatic degradation at high temperatures. Mori *et al*.^25^ also suggested that the loss of anthocyanin in grape berries at high temperatures was due to the non-enzymatic degradation and inhibition of mRNA transcription in anthocyanin biosynthetic genes. It is possible that anthocyanin biosynthesis was down-regulated to save energy due to the ease of its non-enzymatic degradation at high temperatures, or that high temperatures damaged the genes or their transcripts as suggested in previous studies. However, in a recent analysis of the flavonol and anthocyanin concentration as well as the expression of genes related to their metabolism in 54 *Arabidopsis* accessions during cold acclimation, Schulz *et al*.^26^ found only a minor correlation between metabolites and transcripts, and they highlighted the importance of the post-transcriptional regulation of flavonoid metabolism in response to cold. Although the expression of key genes involved in anthocyanin synthesis in the grape berry were decreased, the differences between the expression levels of *VvUFGT* were very small at the different temperatures, and the enzyme activity of UFGT increased at high temperatures in the treated berries^25^ UFGT has been suggested to be the limiting step in anthocyanin synthesis in grape berry, apple, and pear^14, 27, 28^. Changes in gene transcript levels do not always correspond to changes in protein levels or enzyme activity^29, 30^, and the enzyme activity shows a better correlation to the metabolite concentration than to gene expression. The maintenance in plants of a high level of UFGT activity to synthesize anthocyanin that will ultimately be chemically degraded remains puzzling. Clearly, if a high temperature does not inhibit or even increase the anthocyanin synthesis capacity at the enzyme level, the decreased anthocyanin concentration cannot simply be explained by non-enzymatic degradation or the inhibition of gene expression. However, if the degradation is enzymatically controlled, then it would be reasonable for the plant to increase the synthesis capacity of anthocyanin and subsequently degrade the pigment. Anthocyanin is synthesized in the cytosol and transported to vacuoles for storage. Although ROS are produced in mitochondria at high temperatures and spatially separated from synthesized anthocyanin, ROS, especially H_2_O_2_, can diffuse across cell membranes^31, 32^ and enter vacuoles. In vacuoles, there is no superoxide dismutase (SOD), ascorbate peroxidase (APX), catalase, or ascorbate–glutathione cycle enzymes^33^, and ROS scavenging depends mainly on antioxidants such as glutathione, ascorbate and phenolic compounds. In vacuoles, Prx has been suggested to use anthocyanin and other flavonoids as substrates to reduce the H_2_O_2_ that escapes from other organelles^8, 34^. Thus, it is very possible that at high temperatures, plants increase their anthocyanin synthesis capacity and then transfer the anthocyanin into vacuoles to scavenge the diffused ROS. Indeed, even the expression of biosynthetic genes decreased in apple peel following a high temperature treatment, and increased expression of the genes corresponding to anthocyanin transport from the cytosol into the vacuole was observed^35^. Clearly, the metabolic mechanism underlying the decrease in anthocyanin at high temperatures remains unconvincing and requires additional studies for clarification.

In the present study, we used ‘Red Beauty’ plum fruit, which contains an abundance of anthocyanin in both fruit peel and flesh to study anthocyanin metabolism at a high temperature. We chose this material for this study based on three features. First, unlike leaves in which metabolism depends mainly on photosynthesis, fruit can be maintained in the dark for a long time period to exclude the inference of light; second, even in the dark, ‘Red Beauty’ plum can still synthesize abundant anthocyanins in either the fruit peel or flesh. Thus, it is possible to study the metabolism of anthocyanin in the dark. Third, attached or detached ‘Red Beauty’ plum fruit usually becomes red within several days after the veraison stage, which makes it possible to complete the treatment in a short time and preclude the effects of fruit development as much as possible.

## Results

### Phenolic compound concentration

**Figure 1:**
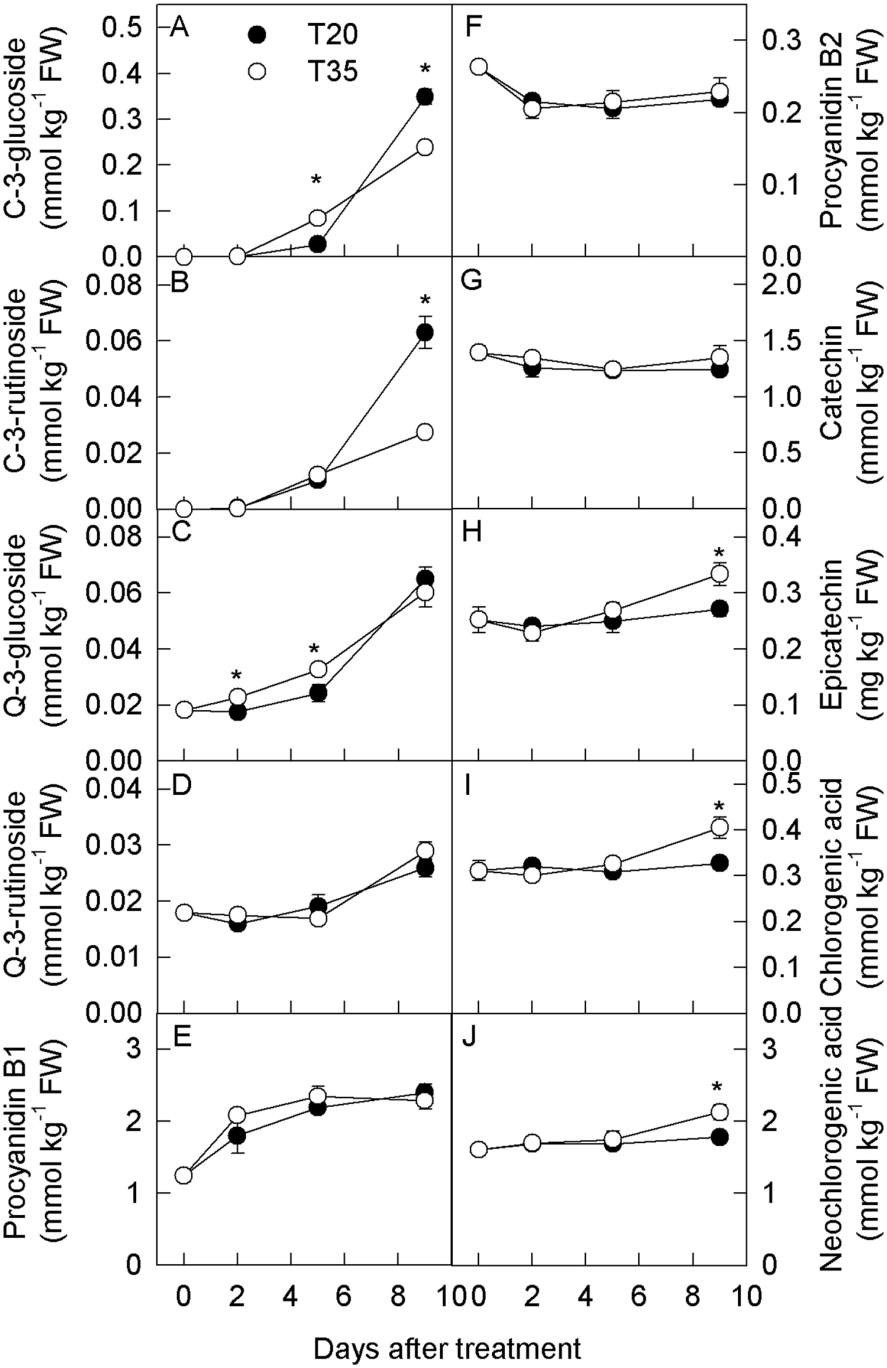
Concentrations of phenolic compounds in the peel of plum fruits treated at 20 °C and 35 °C for different time in the dark. Each data point represents mean ± SE (n = 5). The asterisk indicates a significant difference between two temperature treatments at *P* < 0.05, *t*-test. C-3-glucoside, cyanidin-3-glucoside; C-3-rutinoside, cyanidin-3-rutinoside; Q-3-glucoside,quercetin-3-glucoside; Q-3-rutinoside, quercetin-3-rutinoside. The same as below.

Ten phenolic compounds in ‘Red Beauty’ plum fruit were identified and quantified (Fig. 1). The major anthocyanin components were cyanidin-3-glucoside and cyanidin-3-rutinoside (Supporting information Fig. S1). During treatment, the concentrations of anthocyanins increased in the fruit peel at the two temperatures (Fig. 1A,B). However, in comparison to 20 °C, the concentrations of anthocyanins were significantly higher on day 5 but lower on day 9 at 35 °C. The concentration of quercetin-3-glycoside also increased during the two temperature treatments (Fig. 1C,D). At the higher temperature treatment, the concentrations of quercetin-3-glucoside increased in the peel on days 2 and 5. The concentrations of quercetin-3-rutinoside were comparable at the two temperatures. The concentration of procyanidin B1 increased while that of procyanidin B2 decreased during the treatments (Fig. 1E,F), demonstrating comparable concentrations between the two temperatures. The concentrations of catechin decreased slightly and were comparable during the two temperature treatments (Fig. 1G), and the concentrations of epicatechin hardly changed at 20 °C but increased slightly on day 9 at 35 °C during the treatments, exhibiting a significantly higher concentration at 35 °C compared with 20 °C (Fig. 1H). The concentrations of chlorogenic acid and neochlorogenic acid changed in patterns similar to that of epicatechin (Fig. 1I,J).

The concentrations of anthocyanins as well as other phenolic compounds in the flesh of the fruit changed similarly to those in the peel, except that the concentrations of quercetin-3-glycoside were lower at 35 °C than at 20 °C on day 9 (Supporting information Fig. S2).

### Transcription levels of structural genes and activities of enzymes involved in anthocyanin synthesis

**Figure 2:**
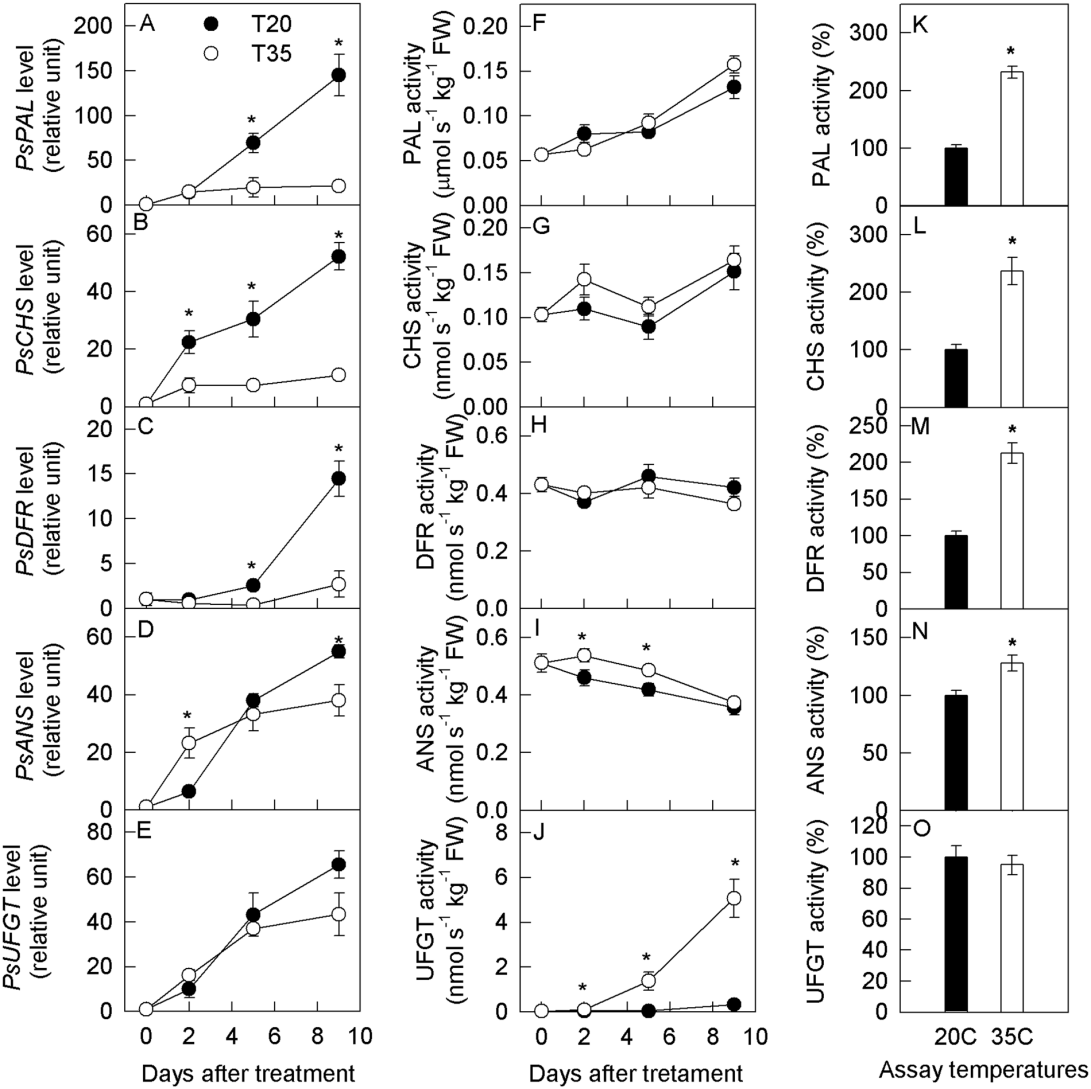
Transcription levels of key genes and the activities of correspondingly encoding enzymes involved in anthocyanin synthesis in the peel of plum fruits treated at 20 °C and 35 °C for different time in the dark. Panel A–E, mRNA level; Panel F–J, enzyme activity assayed at 20 °C; Panel K–O, enzyme activity in 35 °C treated fruit for 9 days assayed at 20 °C and 35 °C. Each data point represents mean ± SE (n = 5). The asterisk indicates a significant difference between two temperature treatments at *P* < 0.05, *t*-test. PAL, phenylalanine ammonia-lyase; CHS, chalcone synthase; DFR, dihydroflavonol reductase; ANS, anthocyanidin synthase; UFGT, UDP glucose:flavonoid 3-O-glucosyltransferase. The same as below.

During treatments, the transcription levels of *PsPAL*, *PsCHS*, *PsDFR*, *PsANS*, and *PsUFGT* increased in the peel of plum fruit (Fig. 2A–E). However, the increases in *PsPAL*, *PsCHS*, and *PsDFR* levels were slower at 35 °C than at 20 °C. The levels of *PsANS* increased at a faster rate on day 2 at 35 °C, but then they were comparable in response to the two temperature treatments on day 5 and eventually decreased at 35 °C on day 9. No differences in the transcription levels of *PsUFGT* were detected between the two temperatures. Similar changes were found in the flesh of the fruit (Supporting information Fig. S3).

The enzyme activities were assayed at 20 °C for fruits that were treated at the two temperatures and also at 35 °C for fruits that were treated at 35 °C, respectively (Fig. 2F–J). When the enzyme activity assays were performed at 20 °C, the activity of PAL increased slightly in the fruit peel during the treatment (Fig. 2F). Moreover, the activity of PAL was comparable between the two temperatures. The activities of CHS and DFR hardly changed and were comparable at the two temperatures (Fig. 2G,H). The activity of ANS decreased slightly during the treatments, but it decreased at a slower rate at 35 °C than that at 20 °C on days 2 and 5 (Fig. 2I). The activity of UFGT increased in response to the treatments (Fig. 2J). However, it increased much faster at 35 °C beginning on day 2. The activities of these five enzymes in the flesh changed in patterns that were similar to those in the peel (Supporting information Fig. S3).

For the fruits treated at 35 °C, the activities of PAL, CHS, DFR, and ANS were significantly higher at 35 °C than at 20 °C (Fig. 2K–N). However, the activity of UFGT was equivalent at the two temperatures (Fig. 2O).

### Respiration rate and ethylene production

**Figure 3:**
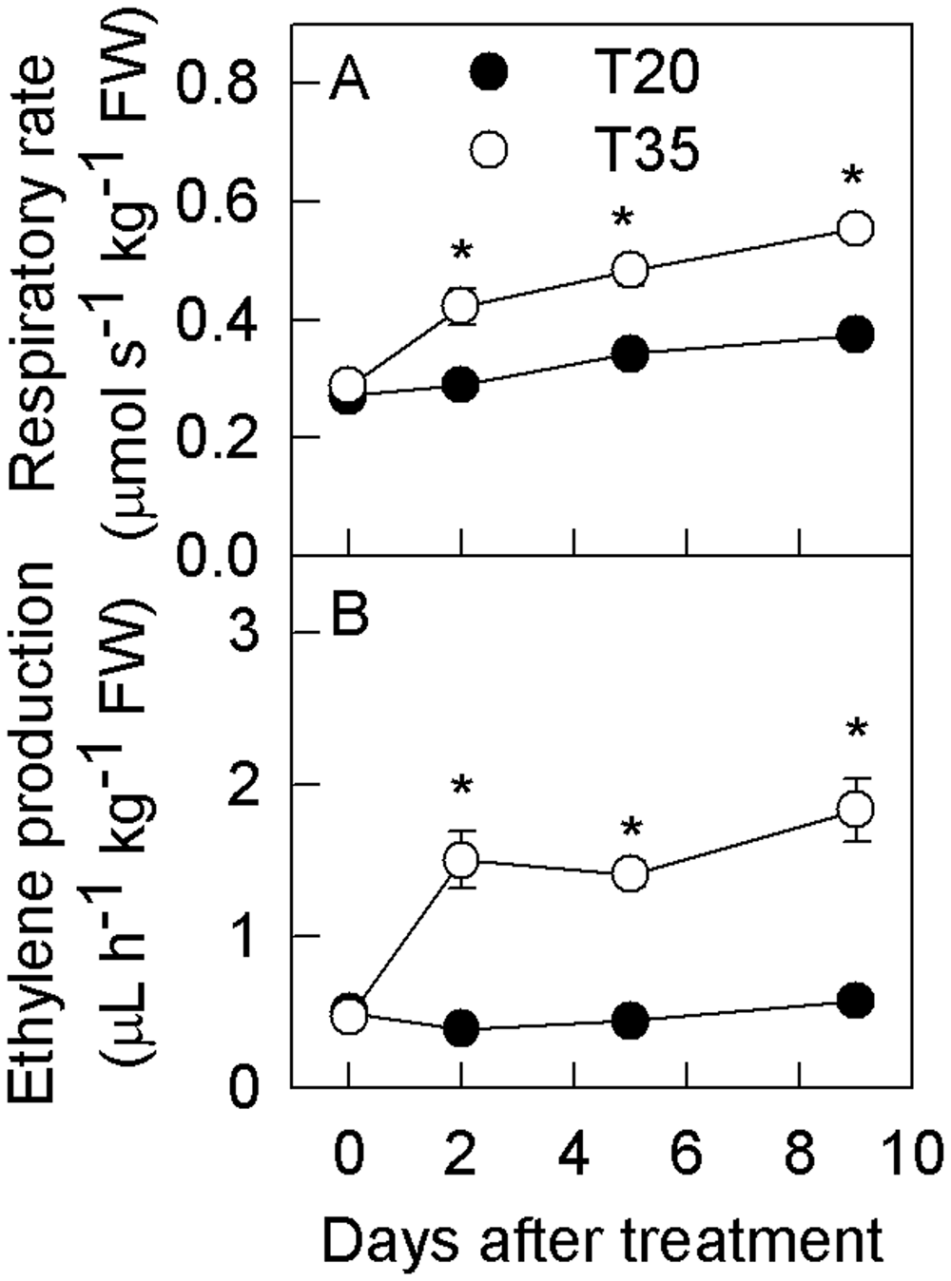
Dark respiration (**A**) and ethylene production (**B**) of plum fruits treated at 20 °C and 35 °C for different time in the dark. Each data point represents mean ± SE (n = 5). The asterisk indicates a significant difference between two temperature treatments at *P* < 0.05, *t*-test.

During the treatments, dark respiration increased in the fruit, whereas the production of ethylene hardly changed at 20 °C (Fig. 3A,B). In comparison to 20 °C, the treatment at 35 °C significantly enhanced the rate of respiration as well as ethylene production in the plum fruit.

### Effects of respiration and ethylene on anthocyanin synthesis

**Figure 4:**
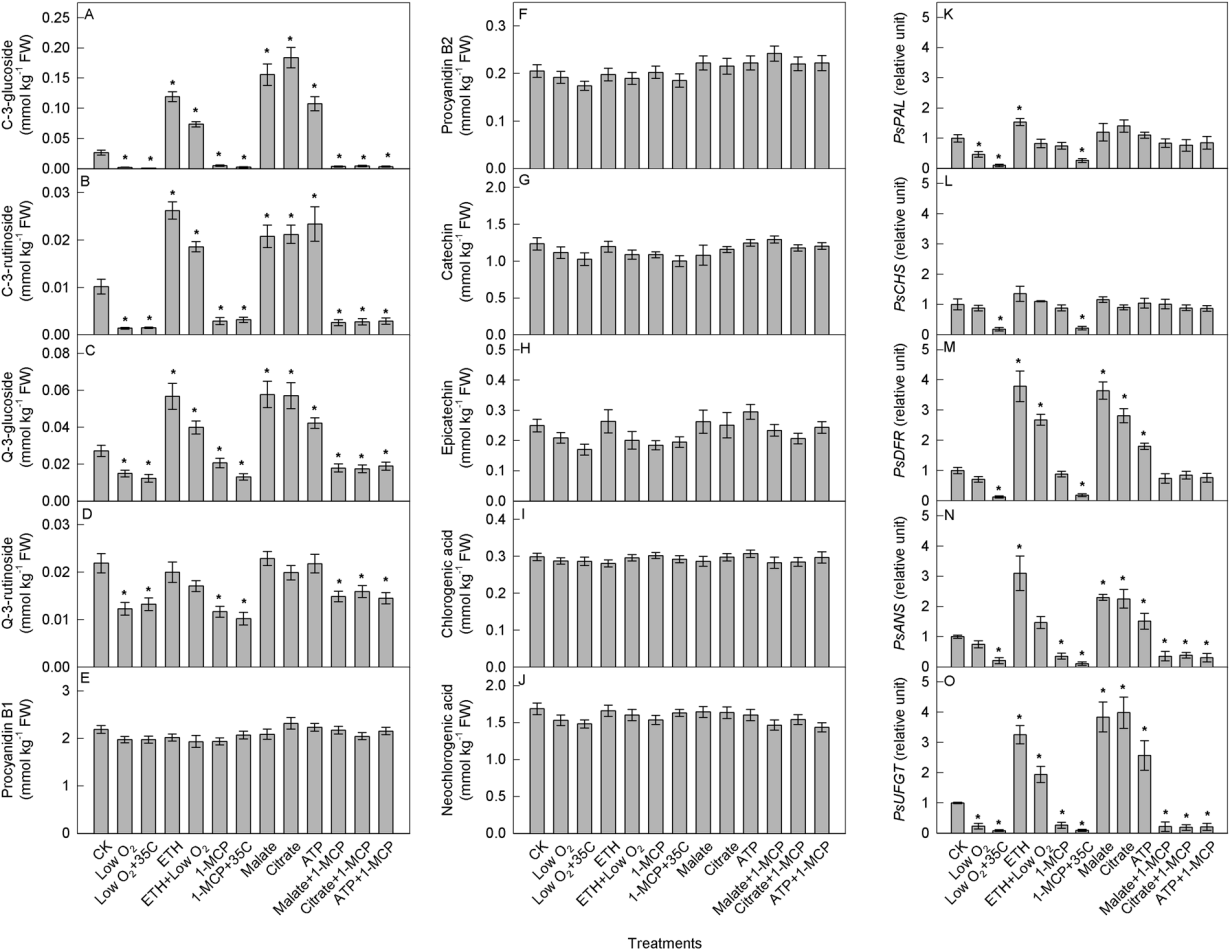
Concentrations of phenolic compounds (**A–J**) and transcript levels of key genes involved in anthocyanin synthesis (**K–O**) in the peel of plum fruits with different treatments in the dark. Each data point represents mean ± SE (n = 5). The asterisk indicates a significant difference between untreated and treated fruits at *P* < 0.05, *t*-test.

To evaluate the mechanism that regulates anthocyanin synthesis in the dark, respiration and the ethylene-dependent signaling pathway were interfered in plum fruits. Considering the diffusion efficiency of chemicals in fruit tissues, only fruit peels were assayed. Among the ten detected phenolic compounds, anthocyanins were the most sensitive compounds to low oxygen, ethylene, 1-MCP^36^ (an inhibitor of ethylene perception), malate, citrate, and ATP treatment alone or in combination (Fig. 4). After five days of treatments, the concentrations of cyanidin-3-glucoside, cyanidin-3-rutinoside, quercetin-3-glucoside, and quercetin-3-rutinoside were significantly reduced under low oxygen or 1-MCP treatment alone at the two temperatures (Fig. 4A–D). Exogenous ethylene, malate, citrate, and ATP significantly increased the concentrations of cyanidin-3-glucoside, cyanidin-3-rutinoside, and quercetin-3-glucoside but did not change the levels of quercetin-3-rutinoside in fruit peel. In comparison to ethylene treatment alone, the combination of ethylene with low oxygen decreased the concentrations of cyanidin-3-glucoside, cyanidin-3-rutinoside, and quercetin-3-glucoside slightly; however, they were still significantly higher than those in the untreated fruit peel. The combined treatment of malate, citrate, or ATP with 1-MCP had similar effects to 1-MCP treatment alone. The exogenous treatments did not change the concentrations of other phenolic compounds.

The transcription levels of *PsPAL* increased slightly in response to exogenous ethylene treatment but did not change following treatment with malate, citrate or ATP (Fig. 4K). Low oxygen or 1-MCP treatment alone decreased the levels of *PsPAL*, especially at higher temperatures. However, ethylene together with low oxygen, or malate, citrate, or ATP combined with 1-MCP, did not change the level of *PsPAL* in the treated fruit peels. The transcript levels of *PsCHS* did not change in response to the different treatments, excluding a decrease in response to low oxygen or 1-MCP treatment at 35 °C (Fig. 4L). Exogenous ethylene, malate, citrate, and ATP treatment alone and ethylene combined with low oxygen increased the level of *PsDFR* approximately 2–4 times (Fig. 4M). Low oxygen or 1-MCP treatment alone did not change the level of *PsDFR* at 20 °C but decreased it at 35 °C. Malate, citrate, or ATP combined with 1-MCP treatment did not change the level of *PsDFR* in comparison to that in untreated fruit. *PsANS* was not affected by low oxygen alone or in combination with ethylene treatment. Ethylene, malate, citrate, or ATP alone increased whereas 1-MCP alone or combined with malate, citrate, or ATP decreased the transcript levels of *PsANS*, especially at 35 °C (Fig. 4N). The levels of *PsUFGT* changed in patterns that were similar to those observed for the concentration of anthocyanin in response to different treatments (Fig. 4O).

### Hydrogen peroxide production and Prx activity and their cytochemical location

**Figure 5:**
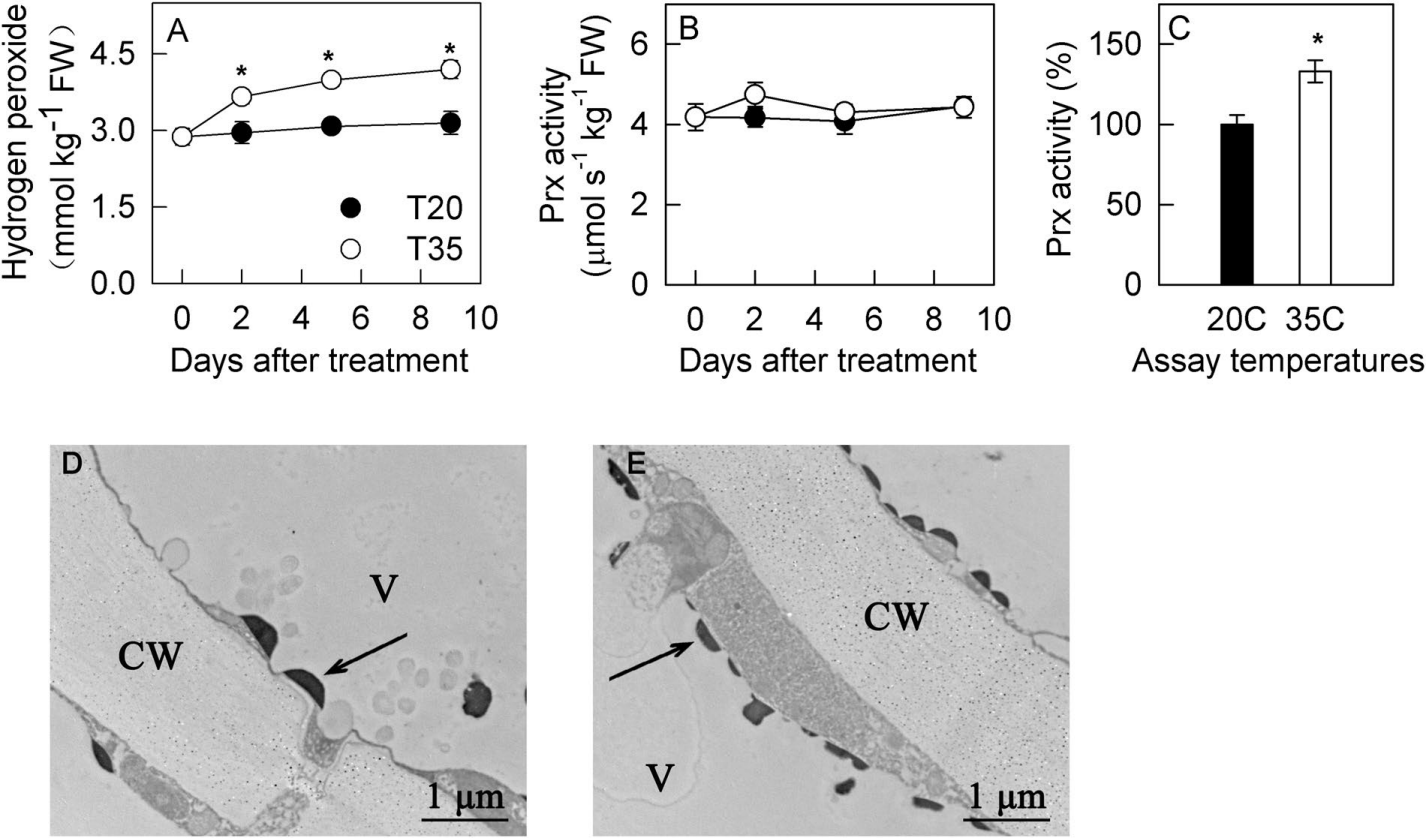
Hydrogen peroxide concentration and class III peroxidase (Prx) activity in the peel of plum fruits treated at 20 °C and 35 °C for different time in the dark. Panel A, hydrogen peroxide concentration in the peels during treatments; Panel B, Prx activity in fruit peels assayed at 20 °C; Panel C, Prx activity in 35 °C treated fruit for 9 days assayed at 20 °C and 35 °C; Panel D and E, cytochemical localization of hydrogen peroxide and Prx activity. Sections were incubated with CeCl_3_, and the electron-dense deposits represent of both Prx activity and produced hydrogen peroxide in the inner part of the tonoplast, marked with arrows. The vacuolar region is marked with “V”. Bar, 1 μm. Each data point represents mean ± SE (n = 5) in Panel A–C. The asterisk indicates a significant difference between two temperature treatments at *P* < 0.05, *t*-test.

During the treatment, the concentrations of hydrogen peroxide in the fruit peel remained unchanged at 20 °C but increased at 35 °C (Fig. 5A). In comparison to 20 °C, the concentration of hydrogen peroxide increased significantly beginning on day 2 in the peel at 35 °C. The concentration of hydrogen peroxide in the fruit flesh also increased on day 9 at 35 °C in comparison to 20 °C (Supporting information Fig. S4).

Prxs are considered to be ubiquitous enzymes in the walls and vacuoles of plant cells, but more than 90% of the total Prx activity is localized in vacuoles^37^. Moreover, determination of the total Prx activity in the extracts led to activity estimations of 96.7–99.7% for the vacuoles and 0.3–3.3% for the cell wall^37^. Because the Prx was extracted according to a protocol similar to that of Ferreres *et al*.^37^, the Prx activity in the present study mainly represented that in vacuoles. When the enzyme activity assay was conducted at 20 °C, the Prx activity in the fruit peel hardly changed and was comparable at the two temperatures (Fig. 5B). The activity of Prx increased in the flesh of the fruit and was higher at 35 °C beginning on day 5 (Supporting information Fig. S4). In the fruits that were treated at 35 °C, increased Prx was detected at 35 °C compared with 20 °C (Fig. 5C).

In previous studies, the cytochemical localization of Prx activity was usually identified by incubating the sections with both exogenous 3,3′-diaminobenzidine (DAB) and hydrogen peroxide. The control was evaluated using the same reaction mixture in the absence of hydrogen peroxide^8, 37^. However, in the present study, CeCl_3_ staining, which is usually used for the cytochemical localization of endogenous hydrogen peroxide in plants^38^, was incubated with the sections without the addition of exogenous hydrogen peroxide. In this way, the cytochemical location of both endogenous hydrogen peroxide and Prx activity in the fruits were performed simultaneously. The results showed that hydrogen peroxide-dependent CeCl_3_ oxidation formed black sediments in the inner region of the tonoplast, especially at 35 °C (Fig. 5D and E, Supporting information Fig. S4), demonstrating the presence of Prx activity associated with the inner face of the vacuolar membrane and endogenous hydrogen peroxide in the vacuoles.

### Enzymatic and non-enzymatic degradations of anthocyanin

**Figure 6:**
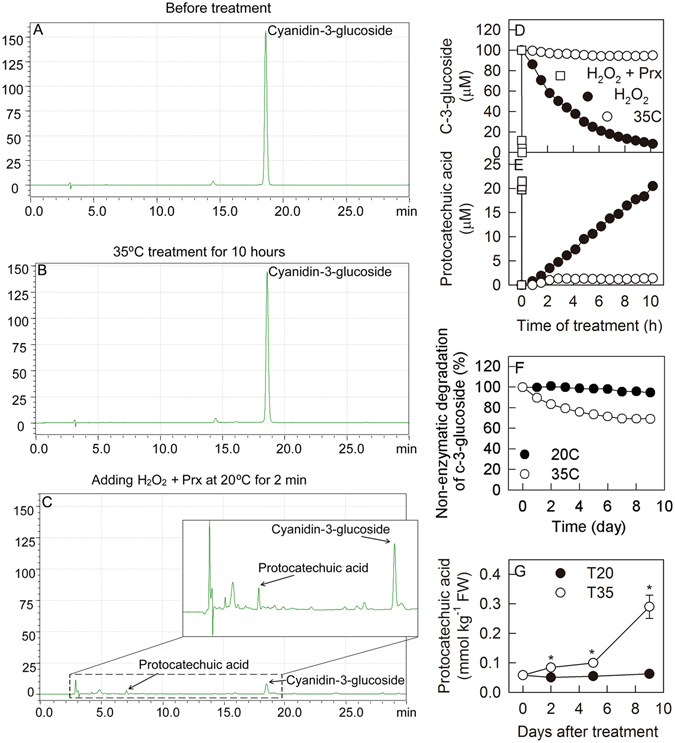
Degradation of anthocyanin. Panel A–C, chromatogram of cyandin-3-glucoside with non-enzymatic or enzymatic degradation induced by hydrogen peroxide; Panel D and E, changes of cyanidin-3-glucoside concentration and production of protocatechuic acid during non-enzymatic degradation or after adding hydrogen peroxide with or without horseradish peroxidase at pH 4.0. Panel F, non-enzymatic degradation of cyanidin-3-glucoside at 20 °C and 35 °C at pH 4.0; Panel G, concentrations of protocatechuic acid in the peel of plum fruits treated at 20 °C and 35 °C for different time in the dark. Each data point represents mean ± SE (n = 5). The asterisk indicates a significant difference between two temperature treatments at *P* < 0.05, *t*-test.

The degradation of anthocyanin by hydrogen peroxide was assessed using cyanidin-3-glucoside solution *in vitro*. Following the addition of hydrogen peroxide to the anthocyanin solution, the degradation of anthocyanin accelerated markedly, especially with the addition of 1 unit of Prx (Fig. 6A–D). Moreover, following the degradation by hydrogen peroxide, some new compounds were detected, including protocatechuic acid (Fig. 6C). Following the addition of hydrogen peroxide, the production of protocatechuic acid increased consistently with the degradation of anthocyanin (Fig. 6D,E). However, the molar production of protocatechuic acid was lower than the molar degradation of anthocyanin.

Using cyanidin-3-glucoside solution, the non-enzymatic degradation of anthocyanin was also compared at 20 °C and 35 °C *in vitro*. The non-enzymatic degradation of anthocyanin increased at 35 °C in comparison to 20 °C (Fig. 6F). After 9 days of treatment, approximately 30% of the anthocyanin was degraded at 35 °C in comparison to only 5% at 20 °C.

During the two temperature treatments, the concentration of protocatechuic acid remained unchanged at 20 °C but increased at 35 °C in both fruit peel and flesh, with a higher value measured at 35 °C beginning on day 2 in the peel and on day 9 in the flesh (Fig. 6G, Supporting information Fig. S5). This result suggested that the degradation of anthocyanin by hydrogen peroxide in plum fruits was increased at 35 °C compared with 20 °C.

**Figure 7:**
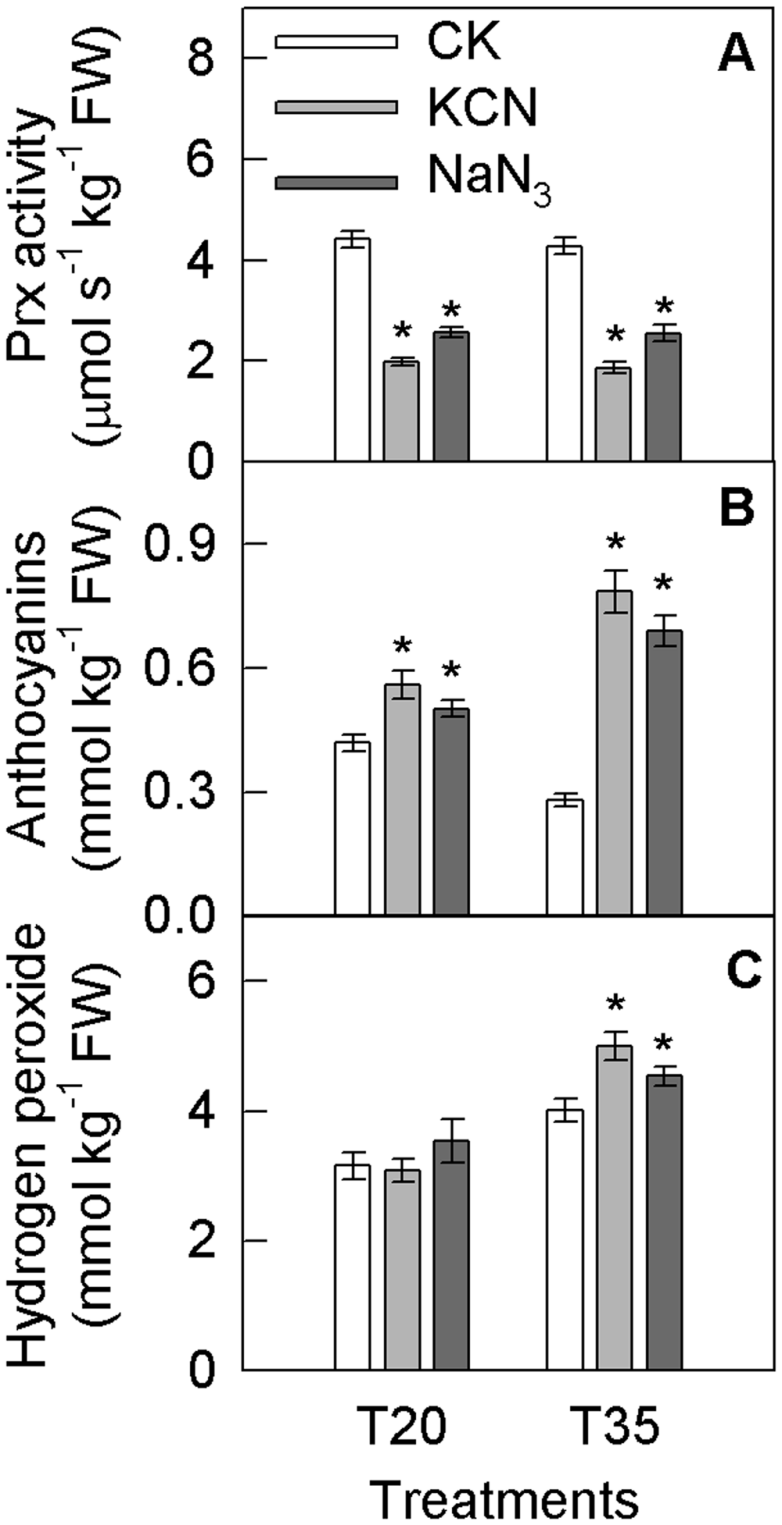
Class III peroxidase (Prx) activities (**A**) and anthocyanin (**B**) and hydrogen peroxide (**C**) concentrations in the peels of plum fruits exposed to different treatment in the dark. Each data point represents mean ± SE (n = 5). The asterisk indicates a significant difference between two temperature treatments at *P* < 0.05, *t*-test.

KCN and NaN_3_ are known to inhibit the activity of Prx^39, 40^. Although these two chemicals might have different other effects on the metabolism in fruits peel, the inhibitions of Prx by KCN and NaN_3_ were both accompanied with the increase in anthocyanin and hydrogen peroxide concentrations, especially at 35 °C after 9 days of treatment (Fig. 7; Supporting information Fig. S6). As a result, the anthocyanin concentration in KCN and NaN_3_ treated fruits became significantly higher at 35 °C than that at 20 °C.

**Figure 8:**
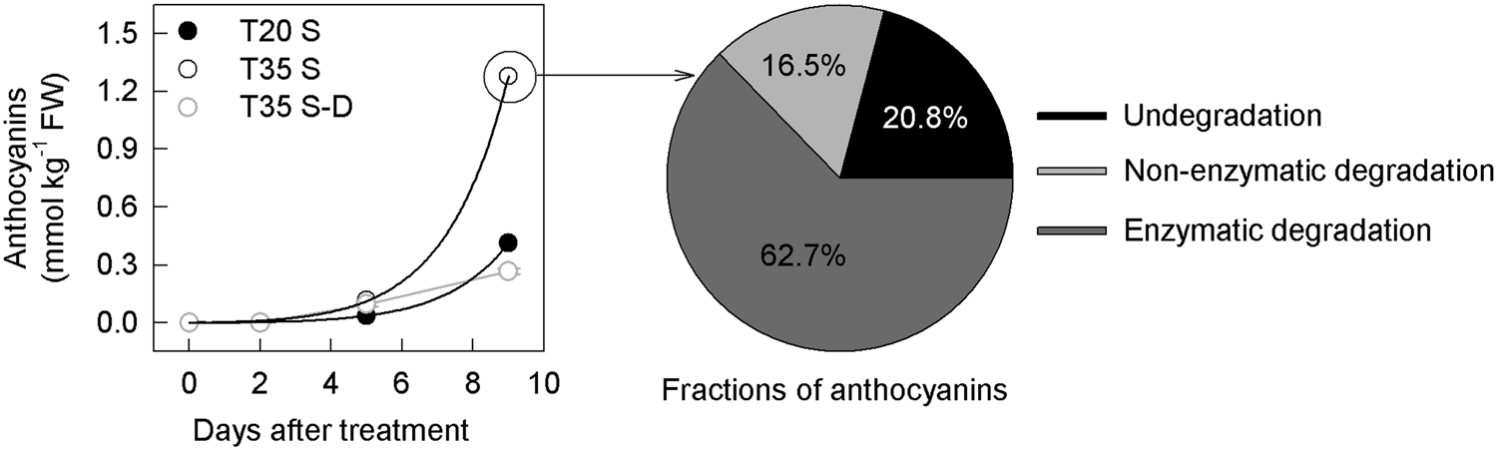
Panel A, concentrations of grossly synthesized anthocyanin in the peel of plum fruits treated at 20 °C and 35 °C for different time in the dark; Panel B, fractions of anthocyanins in 35 °C treated fruit peel at day 9.

To calculate the amount of degraded anthocyanin caused by the increase in temperature from 20 °C to 35 °C, degradation at 20 °C was not considered. Indeed, both the non-enzymatic and enzymatic degradation of anthocyanin were very low at 20 °C (Fig. 6F,G; Supporting information Fig. S5). The formula (9) $A(t)=K\times {t}\times ({e}^{{k}_{3}t})$ could be used to directly fit changes in the time course of anthocyanin concentrations in 20 °C-treated fruits (Fig. 8, Supporting information Fig. S7; for details regarding the formula, please refer to the Materials and methods section). At 20 °C, the following was applied:

1 $$A({\rm{5}})=5{K}_{0}\times ({e}^{-\frac{{\rm{\Delta }}{E}_{a}}{R{T}_{1}}})\times ({e}^{5{{\rm{k}}}_{3}})$$

2 $$A({\rm{9}})=9{K}_{0}\times ({e}^{-\frac{{\rm{\Delta }}{E}_{a}}{R{T}_{1}}})\times ({e}^{9{{\rm{k}}}_{3}})$$

3 $$A(9)/A(5)=\frac{9}{5}\times \frac{{{\rm{e}}}^{9{{\rm{k}}}_{3}}}{{e}^{5{k}_{3}}}$$

Clearly,

4 $${[A(9)/A(5)]}_{20^\circ {\rm{C}}}={[A(9)/A(5)]}_{35^\circ {\rm{C}}}$$

5 $${[A(9)]}_{35^\circ {\rm{C}}}={[A(9)/A(5)]}_{20^\circ {\rm{C}}}\times {[A(5)]}_{35^\circ {\rm{C}}}$$

Thus, ${[A(9)]}_{35^\circ {\rm{C}}}$ could be calculated using formula (5) if ${[A(5)]}_{35^\circ {\rm{C}}}$ was known. Because the levels of protocatechuic acid in fruit flesh were similar at the two temperatures on day 5, we hypothesized that very little anthocyanin was undergoing enzymatic degradation when the temperature increased from 20 °C to 35 °C on day 5. Thus, ${[A(9)]}_{35^\circ {\rm{C}}}$ in the flesh could be calculated using the measured anthocyanin concentration plus that undergoing non-enzymatic degradation at 35 °C on day 5. In fruit peel, a greater amount of protocatechuic acid was produced on day 5 at 35 °C than at 20 °C, which indicated that anthocyanin degradation occurred on day 5 when the temperature increased from 20 °C to 35 °C. Because the production of protocatechuic acid was markedly lower on day 5 than on day 9 (Fig. 6G), given anthocyanin degradation in the peel on day 5 was negligible, ${[A(9)]}_{35^\circ C}$ could be calculated approximately based on an underestimation using the measured anthocyanin concentration together with that undergoing non-enzymatic degradation at 35 °C on day 5. In this way, curve fitting using $A(t)=0.0007t\times {e}^{0.4597t}$ with ${{\rm{R}}}^{2}=0.9999$ for fruit peel and $A(t)=0.0001t\times {e}^{0.5454t}$ with ${{\rm{R}}}^{2}=1$ for flesh could be obtained at 20 °C, and curve fitting using $A(t)=0.0022t\times {e}^{0.4606t}$ with ${{\rm{R}}}^{2}=0.9999$ for peel and $A(t)=0.0005t\times {e}^{0.5454t}$ with ${{\rm{R}}}^{2}=1$ for flesh could be obtained at 35 °C. Clearly, the *k*_3_ value of fruit flesh was the same at the two temperatures. There was a slight difference in the *k*_3_ value of the fruit peel between the two temperatures, which could be derived from an underestimation of ${[A(5)]}_{35^\circ C}$ with the negligible anthocyanin degradation at 35 °C on day 5.

According to the rate of the non-enzymatic degradation of anthocyanin (Fig. 6F), the amount of degraded anthocyanin could be calculated (for example, the amount of anthocyanin via non-enzymatic degradation on day 9, ${A}_{{cd}}\approx ({A}_{(9)}-{A}_{(5)})$ × $[({R}_{c(4)}+{R}_{c(3)}+{R}_{c(2)}+{R}_{c(1)})/4]+{A}_{(5)}$ × $[({R}_{c(5)}+{R}_{c(4)}+{R}_{c(3)}+{R}_{c(2)}+{R}_{c(1)})/5]$, with ${R}_{c(t)}$ denoting the rate of non-enzymatic degradation at time *t*). The fate of the grossly synthesized anthocyanins at 35 °C could then be calculated accordingly. On day 9, 16.5% of the anthocyanin was non-enzymatically degraded as the temperature increased from 20 °C to 35 °C, whereas 62.7% of the anthocyanins in the peel and 72.0% in the flesh were degraded via other processes (enzymatic degradation). The residual anthocyanins represented 20.8% and 11.5% in the peel and flesh, respectively (Fig. 8, Supporting information Fig. S7).

## Discussion

At room temperature in the dark, anthocyanin synthesis in plum fruit was clearly regulated by respiration and ethylene-dependent signaling (Fig. 4). Treatment with 1-MCP alone or in combination with malate/citrate (Fig. 4) revealed that respiration functioned up-stream of the ethylene-dependent signaling in the regulation of anthocyanin synthesis. Although plum is a climacteric fruit, in the present study, the respiratory climacteric did not occur during the treatment (data not shown), indicating that the regulation of anthocyanin synthesis did not relate to the respiratory climacteric. Namely, there was no clear senescence of the fruit during the experiment. Vaknin *et al*.^41^ also suggested that the degradation of anthocyanin was not part of the general senescence process but rather a distinctive and specific pathway. The complete oxygenolysis of one molecule of sucrose can produce 30 or 32 ATP molecules through glycolysis and the TCA cycle. ATP is the key substrate for ethylene production via the methionine cycle in plants^42^. The regulation of ethylene production by respiration has been reported in tomato^43^. Thus, it is possible that dark respiration regulated ethylene production by producing ATP and subsequently regulated anthocyanin synthesis in plum fruit (Fig. 4). In previous studies, positive regulation of anthocyanin synthesis by ethylene has been observed in grape berry^44^, apple^45^, pear^46^, and mangosteen^47^. Our results also showed that the concentration of anthocyanin in plum fruit correlated very well with the transcript level of *PsUFGT* (Fig. 4). UFGT is the limiting step in anthocyanin synthesis in grape berry, apple, and pear^14, 15, 27, 28^. In response to different treatments, the concentrations of the other eight detected phenolic compounds (Fig. 4) provide additional support that UFGT might be the key step in anthocyanin synthesis.

Because high temperatures can enhance the respiratory rate and ethylene production (Fig. 3), it was hypothesized that a high temperature might also increase the gene expression and production of anthocyanins in plum fruit. However, the levels of *PsUFGT* remained unchanged, while the levels of other four genes (*PsPAL, PsCHS, PsDFR*, and *PsANS*) were reduced at 35 °C than at 20 °C on days 5 and 9 (Fig. 2, Supporting information Fig. S3). We speculate that, on the one hand, high temperature enhanced gene expression by increasing respiration and ethylene production. However, on the other hand, high temperature could also directly reduce gene expression in the fruit, as supported by the expression levels detected in response to low oxygen and 1-MCP at the two temperatures (Fig. 4). The eventual transcript levels represented the combined results of the positive and negative effects of high temperature. In previous studies investigating apple peel^35^, grape berry peel^25, 48^, and kiwifruit flesh^49^, the transcript levels of the genes involved in anthocyanin synthesis were reduced at high temperatures, suggesting that the negative effect on gene expression might be stronger than the positive effect of high temperature. Additional studies are needed to clarify this supposition.

Interestingly, the high temperature displayed different regulatory effects on anthocyanin synthesis at the mRNA and enzyme activity levels (Fig. 2, Supporting information Fig. S3). Unlike the mRNA levels, the corresponding enzyme activity was higher in the fruit treated at 35 °C compared with 20 °C (Fig. 2, Supporting information Fig. S3). Enzymes are generated via transcription, translation, modifications and other processes. In comparison to gene expression, the activity of enzymes correlates more closely with the metabolite concentration. Thus, the results of the present study indicate that the synthesis capacity of anthocyanin did not decrease but increased in response to the high temperature treatment. In previous studies, it has been suggested that the synthesis of anthocyanin is inhibited at the mRNA level in grape berries, while the activity of UFGT increases in samples treated at a high temperature^25^. A high-throughput analysis of gene expression and protein levels revealed that the gene transcript levels were not consistent with the levels of their translated proteins^29, 30^. Clearly, high temperature might regulate anthocyanin synthesis in plum fruit at the post-transcriptional or post-translational level, similarly to the phenomenon that occurs in Arabidopsis leaves at low temperature^26^. In addition to anthocyanin, changes in the concentration of the other eight phenolic compounds also suggest that the phenolic synthesis capacity was enhanced at the high temperature (Fig. 1). The concentrations of anthocyanins were higher at 35 °C than at 20 °C on day 5, but an explanation for the lower concentration observed at 35 °C on day 9 remains elusive.

In the present study, although antioxidant enzyme activity was enhanced (Supporting information Fig. S8), the final production of ROS remained higher at 35 °C than at 20 °C (Fig. 5, Supporting information Fig. S4). This finding suggests that the plum fruit suffered from oxidative stress and that the antioxidant system (including SOD, APX, and the ascorbate-glutathione cycle), the first defense against ROS accumulation in mitochondria and the cytosol, could not effectively scavenge the excessive ROS production in a timely manner. The lower reduced/oxidized ascorbate ratios indicated that the capacity of the antioxidant system in mitochondria or cytosol in fruit peels even decreased on days 5 and day 9 (Supporting information Fig. S9). The excess ROS were likely derived from respiratory electron leakage in mitochondria because respiration was significantly enhanced at the high temperature (Fig. 3). Hydrogen peroxide can diffuse across membranes^31, 32^, and therefore, it is very possible that ROS, especially hydrogen peroxide, could diffuse from mitochondria into the vacuole. Indeed, greater amounts of hydrogen peroxide were detected in vacuoles in samples treated at 35 °C compared with 20 °C on day 9 (Fig. 5, Supporting information Fig. S4). Moreover, this phenomenon was accompanied by elevated vacuolar Prx activity (Fig. 5, Supporting information Fig. S4). These results demonstrate that hydrogen peroxide was reduced in vacuoles via catalysis by Prx. Prx can reduce hydrogen peroxide using flavonoids as a substrate^8, 34^. Among the ten evaluated phenolic compounds, anthocyanin exhibited higher antioxidant capacity than the other compounds (Supporting information Fig. S10), which is consistent with our previous studies^50^. In litchi fruit peel, it was reported that the degradation of anthocyanin was catalyzed by an intracellular laccase using epicatechin as a substrate, supported by a dramatic decrease in epicatechin content in the pericarp parallel to anthocyanin degradation^51^. In the present studies, the different change patterns of anthocyanin and epicatechin concentrations (Fig. 1, Supporting information Fig. S2) suggested that laccase might not be involved in anthocyanin degradation in plum fruit. Actually, an inhibition of Prx activity resulting in significantly higher anthocyanin concentration at 35 °C than that at 20 °C also indicated that the enzymatic degradation of anthocyanin in plum fruit was regulated by Prx and much greater at 35 °C (Fig. 7). Even in the absence of Prx catalysis, cyanidin-3-glucoside still showed increased antioxidant capacity compared with reduced ascorbate (Supporting information Fig. S11). Moreover, the activity of GST, which may transfer anthocyanin from the cytosol to the vacuole, was also increased at 35 °C (Supporting information Fig. S12). As a result, it was very possible that anthocyanin reacted with hydrogen peroxide in the vacuoles of plum fruit. Whether β-glucosidase participates in the enzymatic degradation of anthocyanin remains unclear, although cyanidin possesses a higher antioxidant capacity than cyanidin-3-glucoside (Supporting information Fig. S11). Zipor *et al*.^8^ found that in *B. calycina* flowers, β-glucosidase was not involved in anthocyanin degradation. Further studies are needed to clarify the role of β-glucosidase in anthocyanin degradation in plum fruit *in planta*.

Based on an *in vitro* analysis, we identified a product of the reaction between hydrogen peroxide and cyanidin-3-glucoside, procatechuic acid (Fig. 6), which is consistent with previous studies^52, 53^. Interestingly, the non-enzymatic degradation of cyanidin-3-glucoside in response to high temperature did not result in an accumulation of this compound (Fig. 6). Thus, during the treatments, the changes in procatechuic acid also suggest that the enzymatic degradation of anthocyanin induced by hydrogen peroxide *in planta* increased in severity, especially at the high temperature on day 9 (Fig. 6, Supporting information Fig. S5). This phenomenon resulted in a relatively lower anthocyanin concentration in plum fruit at 35 °C on day 9 because the final concentration of any compound in the plants represented the combined results of synthesis and degradation.

Through mathematical modeling, it was estimated that approximately 16.5% of the grossly synthesized anthocyanin was non-enzymatically degraded, while more than 60–70% was degraded via other processes on day 9 when the temperature increased from 20 °C to 35 °C (Fig. 7, Supporting information Fig. S7). The 60–70% of degraded anthocyanin should be mainly related to enzymatic degradation, which was induced by excess hydrogen peroxide.

To the best of our knowledge, this is the first study to reveal that the enzymatic degradation of anthocyanin plays a key role in the determination of fruit color. Plum fruit is a unique system to study anthocyanin synthesis and degradation at high temperatures. At low temperatures in the dark, the synthesis and accumulation of anthocyanin in plum fruit was significantly inhibited (data not shown), in contrast to the results obtained for apple and grape berry peels, in which anthocyanin synthesis was significantly enhanced by low temperature^48, 54^. However, it must be noted that unlike plum fruit, in which light is not essential for the synthesis of anthocyanin, light is essential for the synthesis of anthocyanin in apple and grape berry peels^14, 48, 54^. The different synthesis mechanisms might result in different mechanisms of anthocyanin degradation. However, to comprehensively understand the changes in the concentration of anthocyanin in fruit or other plant tissues, it is more effective to study the synthesis and degradation of anthocyanin at different levels.

## Conclusion

The high temperature applied in this study could enhance the expressions of key genes involved in anthocyanin synthesis by increasing respiration and ethylene production of the plum fruit, but also directly reduce the gene expressions. Although the eventual transcript levels of the genes were reduced at high temperatures, the synthesis capacity of anthocyanin did not decrease but increased in response to the high temperature treatment at the level of enzyme activities. Meanwhile, high temperature enhanced the production of hydrogen peroxide, which triggered the enzymatic degradation of anthocyanin via catalysis by Prx in the vacuoles. Both anthocyanin synthesis and its degradation capacity were enhanced at the high temperature treatment. The anthocyanin concentration in plum fruit depends on the counterbalance of its synthesis and degradation.

## Materials and Methods

### Plant materials and treatments

‘Red Beauty’ plum fruit (*Prunus salicina* Lindl.) was used in the present study. The trees were 6-year-old on wild peach rootstock with an open-center system and were planted with a spacing of 3 m × 4 m in north–south rows in Tai’an (36.13°N, 117.07°E; elevation: 200 m), Shandong province, China. Standard horticultural treatments were applied for disease and pest control. Fruits were harvested at the beginning of the veraison period (the suture of some fruits became red, but most parts of the peel remained green) in June. During fruit development, the maximum light intensity consisted of a photon flux density of 1800 ± 50 μmol m^−2^ s^−1^. The highest air temperature and peel temperature at mid-day was 30 ± 1 and 35 ± 1 °C, respectively, in June.

For the different temperature treatments, the plum fruits were incubated at 20 °C and 35 °C in the dark in growth chambers with a relative humidity of 95%. After 2, 5, and 9 days of treatment, 5 replicates per treatment were analyzed. Ten fruits were collected for each replicate. Fifty fruits were collected before the different temperature treatments (day 0). For the fruit sample collection, the peel and flesh were isolated. Briefly, the fruit peel was obtained with a peeler at a consistent peel thickness of 1 mm, and then the flesh was collected with a knife after removing the stone. All of the samples were placed immediately in liquid nitrogen. Low oxygen treatment was applied by placing the fruits in plastic bags filled with nitrogen gas plus 2–3% air, and the gas was refreshed daily. For the ethylene, malate, citrate, ATP, KCN, and NaN_3_ treatments, the fruits were immersed in 3 mM ethephon, 100 mM malate, 100 mM citrate, 100 mM ATP, 3 mM KCN, or 1 mM NaN_3_ solutions overnight. The fruits were then removed from the solutions and placed in the dark in growth chambers for the temperature treatment. The 1-MCP treatment was conducted by placing the fruits in a sealed container overnight with 1 μL L^−1^ 1-MCP. For the combined malate, citrate, or ATP with 1-MCP treatments, the fruits were treated initially with 1-MCP. Fruits that were immersed overnight in water were used as a control. After 5 days of the ethylene, malate, citrate, ATP, 1-MCP, and the combined treatments or 9 days of the KCN and NaN_3_ treatments at the different temperatures, 5 replicates of the fruit peel samples consisting of 10 fruits per replicate were obtained. The frozen samples were ground to powder using an A11 grinder from IKA^®^ Works (VWR, Radnor, PA, USA) and then stored at −80 °C until analysis.

### Phenolic compound analysis

Phenolic compounds were extracted and analyzed as described by Bi *et al*.^50^ with some modifications. Briefly, the compounds were extracted with 70% methanol containing 2% formic acid at 0–4 °C. After centrifugation at 10,000 g for 10 min, the supernatant was filtered through a 0.22-μm syringe filter prior to HPLC analysis.

Phenolic compounds were analyzed using an LC20A liquid chromatograph equipped with a photo-diode array detector (Shimadzu, Tokyo, Japan). The Inertsil ODS-3 column (5.0-μm particle size, 4.6 mm × 250 mm, GL Sciences Inc., Tokyo, Japan) was used for the separation preceded by an Inertsil ODS-3 Guard Column (5.0 μm, 4.0 mm × 10 mm). Solvent A consisted of 10% formic acid dissolved in water; solvent B was 10% formic acid and 1.36% water in acetonitrile. The gradient was 95% A (0 min), 85% A (25 min), 78% A (42 min), 64% A (60 min), and 95% A (65 min), and the post-run-time was 10 min. The flow rate was 1.0 mL/min at 30 °C. Simultaneous monitoring was performed at 280 nm for procyanidin B1, catechin, procyanidin B2, epicatechin, and protocatechuic acid; at 320 nm for neochlorogenic acid and chlorogenic acid; at 365 nm for quercetin-3-glucoside and quercetin-3-rutinoside; and at 520 nm for cyanidin-3-glucoside and cyanidin-3-rutinoside. Peaks were identified by comparing the retention time and spectra with authentic standards. The concentration of individual phenolic compounds was determined based on the peak area and calibration curves derived from the corresponding authentic phenolic compounds. All of the standards were obtained from Sigma–Aldrich (St. Louis, MO, USA) and Extrasynthese (Genay Cedex, France).

### Real-time qPCR assay

Total RNA was isolated using the sodium dodecyl sulfate–phenol method according to Malnoy *et al*.^55^. First-strand cDNA was synthesized using the PrimeScript^TM^ RT reagent Kit (Takara, Dalian, China) according to the manufacturer’s protocol. Real-time polymerase chain reaction (PCR) was performed with the iQ5 Multicolor Real-Time PCR Detection System (Bio-Rad Laboratories, Hercules, CA) and SYBR Green Master Mix (SYBR Premix EX Taq^TM^, Dalian, China). *PsACTIN* was used to standardize the cDNA samples for different genes. The primers for *PsPAL*, *PsCHS*, *PsDFR*, *PsANS*, and *PsUFGT* are listed in Supporting information Table S1. For each gene assay, three biological replicates were evaluated.

### Enzyme activity assay

For the phenylalanine ammonia-lyase (PAL, EC 4.1.3.5), chalcone synthase (CHS, EC 2.3.1.74), dihydroflavonol reductase (DFR, EC 1.1.1.219), anthocyanidin synthase (ANS, EC 1.14.11.19), UDP glucose:flavonoid 3-O-glucosyltransferase (UFGT, EC 2.4.1.91), and class III peroxidase (Prx, EC 1.11.1.7) extraction, 1.0 g of frozen tissue was ground in 1.8 mL of 100 mM Tris–HCl buffer (pH 7.0) containing 14 mM β-mercaptoethanol, 5 mM DTT, 1% BSA, and 5% PVPP. The enzyme extracts were desalted by passage through PD10 columns and then used immediately for the enzyme analysis.

PAL activity was assayed according to the method of Levis *et al*.^56^ with some modifications. The assay mixture (1.0 mL) contained 100 mM Tris-HCl (pH 8.8), 100 μL of enzyme extract and 10 mM L-phenylalanine. An increase at 290 nm was monitored. The extinction coefficient was 17.4 mM^−1^ cm^−1^.

CHS activity was assayed as described by Alokam *et al*.^57^ with some modifications. The assay mixture contained 200 μL of the enzyme extract, 10 μL of 0.4 mM malonyl CoA, and 10 μL of 0.2 mM 4-coumaroyl CoA. After incubation for 1 hour at 20 or 35 °C, the reaction mixture was extracted twice with 1 mL of ethyl acetate. The ethyl acetate fraction was evaporated until dry under a stream of nitrogen gas. The residue was then dissolved in 100 μL MeOH, and the amount of produced naringenin was analyzed by HPLC at 290 nm.

DFR activity was assayed according to the method of Stafford & Lester^58, 59^ with minor modifications. The incubation mixture contained 300 μmol (±) dihydroquercetin, 20 μmol NADPH, 1 mM DTT, 0.6 mL of the enzyme extract, and an NADPH-regenerating system consisting of 5 units of glucose-6-phosphate dehydrogenase and 0.1 mM of glucose-6-phosphate. The assay mixture was incubated at 20 °C or 35 °C for 1 hour and then extracted twice with 1.0 mL of ethyl acetate. The ethyl acetate fraction was evaporated to dryness under a stream of nitrogen gas. The numbers of molecules of leucocyanidin in the ethyl acetate fraction were estimated based on the acid-catalyzed conversion of leucocyanidin to cyanidin, which was accomplished by the addition of 1 mL of butanol-HCl (95:5 v/v) reagent to the evaporated ethyl acetate fraction and heating at 95 °C for 15 min. The absorbance at 550 nm was monitored. The extinction coefficient was 34.7 mM^−1^ cm^−1^. Control assays were conducted using complete assays with heat-inactivated enzyme.

The ANS activity underlying the production of cyanidin from leucocyanidin was determined according to Pang *et al*.^60^ with some modifications. The reaction mixture (0.5 mL) contained 100 mM KPO_4_ buffer (pH 7.0), 200 mM NaCl, 10 mM maltose, 5 mM DTT, 4 mM sodium ascorbate, 1 mM 2-oxoglutaric acid, 0.4 mM FeSO_4_, 0.1 mM 3,4-cis-leucocyanidin, and 0.4 mL of enzyme extract. After incubation at 20 °C or 35 °C for 1 h, the reaction was terminated by the addition of 50 μl of 36% TCA. The reaction mixture was extracted with 1.0 mL of ethyl acetate. The ethyl acetate fraction was evaporated to dryness under a stream of nitrogen gas. The residue was then dissolved in 100 μL of MeOH and subjected to HPLC analysis at 520 nm.

UFGT was assayed according to Zhang *et al*.^28^ with some modifications. The reaction mixture (200 μL) contained 100 mM Tris-HCl (pH 8.0), 2 mM dithiothreitol, 5 mM UDP-glucose, 0.3 mM cyanidin chloride, and 100 μL of enzyme extract. After incubation for 10 min at 20 °C or 35 °C, the reaction was terminated by addition of 50 μL of 36% TCA. The amount of produced cyanidin-3-glucoside was analyzed by HPLC at 520 nm.

The activity of Prx was assayed by monitoring the increase in absorbance at 470 nm due to guaiacol oxidation^61^ (extinction coefficient of 26.8 mM^−1^ cm^−1^). The reaction mixture (1 mL) contained 100 mM sodium phosphate buffer (pH 7.0), 10 mM H_2_O_2_, 10 mM guaiacol, and 20 µL of enzyme extract. The reaction was initiated by the addition of H_2_O_2_.

### Dark respiration and ethylene production assay

Twenty plum fruits (ten at each temperature) were used for dark respiration and ethylene production throughout the entire experiment. Fruit respiration in the dark was measured using an open system for CO_2_ analysis, a CIRAS-2 gas exchange system (PP Systems, Amesbury, MA, USA) equipped with a customer-made container (volume: 80 mL) that could contain two plum fruits. After completing the respiration analysis, ethylene production was assayed using a portable ethylene analyzer (maximum range: 10 μL/L, resolution, 0.01 μL/L; Empaer Technology, Shenzhen, China) equipped with a customer-made container (volume: 200 mL). Two fruits were sealed in the container for 1 hour, and then the upper phase gas was assayed.

### Hydrogen peroxide concentration assay and cytochemical localization of hydrogen peroxide and peroxidase-like activity

Hydrogen peroxide concentrations were assayed as described by Chen *et al*.^62^. For the cytochemical localization, hydrogen peroxide and Prx activity were visualized at the subcellular level by staining with CeCl_3_^37, 38^. Briefly, day 9 plum fruit peel or flesh were cut into 1-mm strips and incubated in freshly prepared 5 mM CeCl_3_ in 50 mM MOPS at pH 7.2 for 1 hour. The fruit sections were then fixed in 1.25% (v/v) glutaraldehyde and 1.25% (v/v) paraformaldehyde in 50 mM sodium cacodylate buffer, pH 7.2, for 1 hour. After fixation, the tissues were washed twice for 10 min in the same buffer and postfixed for 45 min in 1% (v/v) osmium tetroxide and then dehydrated in a graded ethanol series (30–100%; v/v) and embedded in Eponaraldite (Agar Aids). After 12 hours in pure resin followed by an exchange for fresh resin for 4 hours, the samples were polymerized at 60 °C for 48 hours. The samples were analyzed using a HT7700 transmission electron microscope (Hitachi, Tokyo, Japan).

### Assay to Measure the Gross Amounts of Synthesized Anthocyanin

Based on the correlations between the anthocyanin concentrations and transcript levels of key genes involved in anthocyanin synthesis (Fig. 4), PAL, CHS, DFR, and ANS were excluded from the limiting-step of anthocyanin synthesis in plum fruit. According to the changes in anthocyanin concentration and enzymes activity during the treatments (Figs 1 and 2), we propose that at any time (*t*), the rate of gross anthocyanin (A) synthesis is proportional to the activity of the enzyme UFGT (E). Thus,

6 $$dA/dt={k}_{1}\times {\rm{E}}$$

where *k*_1_ is a rate constant.

The content of A at any moment *t*_1_ can be calculated as follows:

7 $$A(t)={k}_{1}\times {\rm{E}}\times {t}_{1}$$

Based on our experimental results (Fig. 2J, Supporting information Fig. 3J), the values of E *in vitro* represent an exponential function of time,

8 $${\rm{E}}={k}_{2}\times ({e}^{{k}_{3}t})$$

where *k*_2_ is a rate constant reflecting the initial enzyme activity at time zero (*t* = 0), and *k*_3_ is a time constant reflecting the rate of the increase in enzyme activity.

Given that the real activity of E *in vivo* was also an exponential function of time as observed *in vitro*, then the content of A can be described by the following:

9 $$A(t)=K\times {t}\times ({e}^{{k}_{3}t})$$

where *K* is a rate constant reflecting the initial rate of A synthesis.

10 $$K={k}_{1}\times {k}_{2}$$

The initial rate *K* is a parameter that can express the temperature effect of A formation. The enzymatic processes typically display an exponential dependence on the temperature:

11 $$K(T)={K}_{0}\cdot ({e}^{-\frac{{\rm{\Delta }}{E}_{a}}{RT}})$$

Here, *K*(*T*) reflects the dependence of the chemical reaction (A synthesis) on the temperature (*T* – absolute temperature). *K*_0_ is a frequency factor or temperature-independent part of the rate, R is a gas constant, and *E*_a_ (=Δ*G*_0_) is the activation energy (free energy) of the rate-limiting reaction. Thus, formula (9) could be changed to the following:

12 $$A({t})={K}_{0}\cdot ({e}^{-\frac{{\rm{\Delta }}{E}_{a}}{RT}})\times t\times ({e}^{{k}_{3}t})$$

Given that the initial concentration A is not 0, the above formula (12) can be updated to the following:

13 $$A(t)={A}_{0}+K\times {t}\times ({e}^{{k}_{3}t})$$

Or

14 $$A({\rm{t}})={A}_{0}+{K}_{0}\cdot ({e}^{-\frac{\Delta {E}_{a}}{RT}})\times t\times ({e}^{{k}_{3}t})$$

### Statistical Analysis

All of the data were statistically analyzed using the *t*-test at *P* < 0.05 with SPSS 16.0 (SPSS Inc. Chicago, IL, USA).

## Electronic supplementary material

supplementary data

## References

[1] Harborne, J. B. Flavonoids: Distribution and contribution to plant colour. *In Chemistry and Biochemistry of* Plant *Pigments* (ed Goodwin, T. W.) 247–278 (Academic press, 1965).

[2] Smillie RM, Hetherington SE (1999). Photoabatement by anthocyanin shields photosynthetic systems from light stress. Photosynthetica..

[3] Steyn WJ, Wand SJE, Holcroft DM, Jacobs G (2002). Anthocyanins in vegetative tissues: a proposed unified function in photoprotection. New Phytol..

[4] Li P, Castagnolib S, Cheng L (2008). Red ‘Anjou’ pear has a higher photoprotective capacity than green ‘Anjou’. Physiol. Plant..

[5] Gould KS, Mckelvie J, Markham KR (2002). Do anthocyanins function as antioxidants in leaves? Imaging of H_2_O_2_ in red and green leaves after mechanical injury. Plant Cell. Environ..

[6] Neill SO, Gould KS (2003). Anthocyanins in leaves: Light attenuators or antioxidants. Funct. Plant Biol..

[7] Nakabayashi R (2014). Enhancement of oxidative and drought tolerance in Arabidopsis by overaccumulation of antioxidant flavonoids. Plant J..

[8] Zipor G, Duarte P, Carqueijeiro I, Shahar L (2015). In planta anthocyanin degradation by a vacuolar class III peroxidase in *Brunfelsia calycina* flowers. New Phytol..

[9] Winkel-Shirley B (1999). Evidence for enzyme complexes in the phenylpropanoid and flavonoid pathways. Physiol. Plant..

[10] Tanaka Y, Sasaki N, Ohmiya A (2008). Biosynthesis of plant pigments: anthocyanins, betalains and carotenoids. Plant J..

[11] Jaakola L (2013). New insights into the regulation of anthocyanin biosynthesis in fruits. Trends in Plant Science..

[12] Oren-Shamir M (2009). Does anthocyanin degradation play a significant role in determining pigment concentration in plants?. Plant Sci..

[13] Li P, Cheng L (2008). The shaded side of apple fruit becomes more sensitive to photoinhibition with fruit development. Physiol. Plant..

[14] Li P, Ma F, Cheng L (2013). Primary and secondary metabolism in the sun-exposed peel and the shaded peel of apple fruit. Physiol. Plant..

[15] Li P, Zhang Y, Einhornc TC, Cheng L (2014). Comparison of phenolic metabolism and primary metabolism between green ‘Anjou’ pear and its bud mutation, red ‘Anjou’. Physiol. Plant..

[16] Hoch WA, Zeldin EL, McCown BH (2001). Physiological significance of anthocyanins during autumnal leaf senescence. Tree Physiol..

[17] Hughes NM, Morley CB, Smith WK (2007). Coordination of anthocyanin decline and photosynthetic maturation in juvenile leaves of three deciduous tree species. New Phytol..

[18] Hughes NM (2014). Photosynthetic costs and benefits of abaxial versus adaxial anthocyanins in *Colocasia esculenta* ‘Mojito’. Planta.

[19] Hughes NM, Neufeld HS, Burkey KO (2005). Functional role of anthocyanins in high-light winter leaves of the evergreen herb *Galax urceolata*. New Phytol..

[20] Davidson JF, Schiestl RH (2001). Mitochondiral respiratory electron carriers are involved in oxidative stress during heat stress in *Saccharomyces cerevisiae*. Mol. Cell. Biol..

[21] Medina-Silva R (2006). Heat stress promotes mitochondrial instability and oxidative responses in yeast deficient in thiazole biosynthesis. Res. Microbiol..

[22] Murata N, Los DA (1997). Membrane fluidity and temperature perception. Plant Physiol..

[23] Li P, Cheng L (2009). The elevated anthocyanin level in the shaded peel of ‘Anjou’ pear enhances its tolerance to high temperature under high light. Plant Sci..

[24] Rowan DD (2009). Environmental regulation of leaf colour in red 35S: Arabidopsis thaliana. New Phytol..

[25] Mori K, Goto-Yamamoto N, Kitayama M, Hashizume K (2007). Loss of anthocyanins in red-wine grape under high temperature. J. Exp. Bot..

[26] Schulz E, Tohge T, Zuther E, Fernie AR, Hincha DK (2015). Natural variation in flavonol and anthocyanin metabolism during cold acclimation in Arabidopsis thaliana accessions. Plant Cell. Environ..

[27] Boss PK, Davies C, Robinson SP (1996). Analysis of the expression of anthocyanin pathway genes in developing *Vitis vinifera* L. cv Shiraz grape berries and the implications for pathway regulation. Plant Physiol..

[28] Zhang J (2014). Reactive oxygen species produced via plasma membrane NADPH oxidase regulate anthocyanin synthesis in apple peel. Planta..

[29] Griffin TJ (2002). Complementary profiling of gene expression at the transcriptome and proteome levels in *Saccharomyces cerevisiae*. Mol. Cell. Proteomics..

[30] Lu P, Vogel C, Wang R, Yao X, Marcotte EM (2007). Absolute protein expression profiling estimates the relative contributions of transcriptional and translational regulation. Natural Biotechnol..

[31] Yamasaki H, Sakihama Y, lkehara N (1997). Flavonoid-peroxidase reaction as a detoxification mechanism of plant cells against H_2_O_2_. Plant Physiol..

[32] Mubarakshina MM (2010). Production and diffusion of chloroplastic H_2_O_2_ and its implication to signalling. J. Exp. Bot..

[33] Mittler R, Vanderauwera S, Gollery M, Van Breusegem F (2004). Reactive oxygen gene network of plants. Trends in Plant Science.

[34] Agati G, Azzarello E, Pollastri S, Tattini M (2012). Flavonoids as antioxidants in plants: Location and functional significance. Plant Sci..

[35] Lin-Wang K (2011). High temperature reduces apple fruit colour via modulation of the anthocyanin regulatory complex. Plant Cell. Environ..

[36] Sisler EC, Serek M (1997). Inhibitors of ethylene responses in plants at the receptor level: recent developments. Physiol. Plantarum..

[37] Ferreres F (2011). Identification of phenolic compounds in isolated vacuoles of the medicinal plant *Catharanthus roseus* and their interation with vacuolar class III peroxidase: an H_2_O_2_ affair?. J. Exp. Bot..

[38] Bestwick CS, Brown IR, Bennett MH, Mansfield JW (1997). Localization of hydrogen peroxide accumulation during the hypersensitive reaction of lettuce cells to *Pseudomonas syringae* pv *phaseolicola*. Plant Cell..

[39] Ranieri A (2003). Early production and scavenging of hydrogen peroxide in the apoplast of sunflower plants exposed to ozone. J. Exp. Bot..

[40] Šukalović VH, Veljović-Jovanović S, Maksimović JD, Maksimović V, Pajić Z (2010). Characterisation of phenol oxidase and peroxidase from maize silk. Plant Biol..

[41] Vaknin H (2005). Active anthocyanin degradation in *Brunfelsia calycina* (yesterday–today–tomorrow) flowers. Planta..

[42] Yang SF, Hoffman NE (1984). Ethylene biosynthesis and its regulation in higher plant. Annu. Review Plant Physiol..

[43] Xu F, Yuan S, Zhang DW, Lv X, Lin HH (2012). The role of alternative oxidase in tomato fruit ripening and its regulatory interaction with ethylene. J. Exp. Bot..

[44] El-Kereamy A (2003). Exogenous ethylene stimulates the long-term expression of genes related to anthocyanin biosynthesis in grape berries. Physiol. Plantarum..

[45] Blanpied GD (1975). Use of ethephon to stimulate red color without hastening ripening of ‘McIntosh’ apples. J. Am. Soc. Hortic. Sci..

[46] MacLean DD, Murr DP, DeEll JR, Mackay AB, Kupferman EM (2007). Inhibition of PAL, CHS, and ERS1 in ‘Red Anjou’ pear (*Pyrus communis* L.) by 1-MCP. Postharvest Biol. Tec..

[47] Palapol Y, Ketsa S, Lin-Wang K, Ferguson IB, Allan AC (2009). A MYB transcription factor regulates anthocyanin biosynthesis in mangosteen (*Garcinia mangostana* L.) fruit during ripening. Planta..

[48] Azuma A, Yakushiji H, Koshita Y, Kobayashi S (2012). Flavonoid biosynthesis-related genes in grape skin are differentially regulated by temperature and light conditions. Planta..

[49] Man YP (2015). High-temperature inhibition of biosynthesis and transportation of anthocyanins results in the poor red coloration in red-fleshed *Actinidia chinensis*. Physiol. Plantarum..

[50] Bi X (2014). Anthocyanin contributes more to hydrogen peroxide scavenging than other phenolics in apple peel. Food Chem..

[51] Fang F (2015). An intracellular laccase is responsible for epicatechin-mediated anthocyanin degradation in litchi fruit pericarp. Plant Physiol..

[52] Seeram NP, Bourquin LD, Nair MG (2001). Degradation products of cyanidin glycosides from tart cherries and their bioactivities. Journal of Agricultural and Food Chem..

[53] Zhang Y, Sun J, Hu X, Liao X (2010). Spectral alteration and degradation of cyanidin-3-glucoside exposed to pulsed electric field. Journal of Agricultural and Food Chem..

[54] Xie XB (2012). The bHLH transcription factor MdbHLH3 promotes anthocyanin accumulation and fruit colouration in response to low temperature in apples. Plant Cell. Environ..

[55] Malnoy M, Reynoird JP, Mourgues F (2001). A method for isolating total RNA from pear leaves. Plant Mol. Biol. Rep..

[56] Levis CE, Walker JRL, Lancaster JE, Conner AJ (1998). Light regulation of anthocyanin, flavonoid and phenolic acid biosynthesis in potato minitubers *in vitro*. Aust. J. Plant Physiol..

[57] Alokam S, Li Y, Li W, Chinnappa CC, Reid DM (2002). Photoregulation of phenylalanine ammonia-lyase (PAL) and chalcone synthase (CHS) in the accumulation of anthocyanin in alpine and prairie ecotypes of *Stellaria longipes* under varied R/FR. Physiolo. Plantarum..

[58] Stafford HA, Lester HH (1982). Enzymic and nonenzymic reduction of (+)-dihydroquercetin to its 3,4,-Diol. Plant Physiol..

[59] Stafford HA, Lester HH (1984). Flavan-3-ol Biosynthesis. The conversion of (+)-dihydroquercetin and flavan-3,4-*cis*-diol (leucocyanidin) to (+)-catechin by reductases extracted from cell suspension cultures of Douglas Fir. Plant Physiol..

[60] Pang Y, Peel GJ, Wright E, Wang Z, Dixon RA (2007). Early steps in proanthocyanidin biosynthesis in the model legume *Medicago truncatula*. Plant Physiol..

[61] Hammerschmidt R, Nuckles EM, Kuc J (1982). Association of enhanced peroxidase activity with induced systemic resistance of cucumber to *Colletotrichum lagenarium*. Physiol. Plant Pathol..

[62] Chen C, Li H, Zhang D, Li P, Ma F (2013). The role of anthocyanin in photoprotection and its relationship with the xanthophyll cycle and the antioxidant system in apple peel depends on the light conditions. Physiol. Plantarum..

