# Supplementary material for: Anthocyanin concentration depends on the counterbalance between its synthesis and degradation in plum fruit at high temperature
Source: Sci Rep. 2017 Aug 9;7:7684. doi: 10.1038/s41598-017-07896-0 (PMC5550432; doi:10.1038/s41598-017-07896-0)
Supplement: Supplementary file 1 — supplementary data [file 41598_2017_7896_MOESM1_ESM.pdf]

Anthocyanin concentration depends on the counterbalance between its synthesis and degradation in plum fruit at high temperature

Junping Niu[1,#], Guojing Zhang[1,#], Wenting Zhang[1], Vasilij Goltsev[2], Shan Sun[3], Jinzheng Wang[3], Pengmin Li[1,*] & Fengwang Ma[1]

[1]*State Key Laboratory of Crop Stress Biology for Arid Areas, College of Horticulture, Northwest A&F University, Yangling, Shaanxi 712100, China*

2*Department of Biophysics and Radiobiology, Faculty of Biology, St. Kliment Ohridski University of Sofia, 8 Dr. Tzankov Blvd., 1164 Sofia, Bulgaria*

[3]*Shandong Institute of Pomology, Taian, Shandong 271000, China*

[#]They contributed equally to this work.

[*]Corresponding author, Tel. +86 29 87082648; E-mail: Lipm@nwsuaf.edu.cn; Postal address: Taicheng Rd. No. 3, College of Horticulture, Northwest A&F University, Yangling, Shaanxi, 712100, China.

Supplemental Table 1. Primers for quantitative Real-time RT-PCR.

Gene identifier	Gene	Forward primer	Reverse primer
EF585293	*PsActin*	GCAGACAGGATGAGCAAGGAGATTAC	TCTGTTGGAAGGTACTGAGGGATG
KT601054	*PsPAL*	CCTCCCACAGAAGAACAAAGCAAG	GCCTGACTCTTTCGTGCTCCC
KT597917	*PsCHS*	GCGGACTACCAGCTCACCAAG	CACACAACAAGAACACGAGCAC
KT597918	*PsDFR*	GGCTGACCTGGCGGACGAG	CACTTCGTTCTCGGGGTCTTTGG
KT601053	*PsANS*	GAGTACATCAGACCCAAGGAAGAGC	GCCTTCTTCAACTCCTCCCTGC
KT597919	*PsUFGT*	CTCCATCAAGCCTAAACTCTCCC	ATTGGTGGTGTGGTAGTGTGGTG

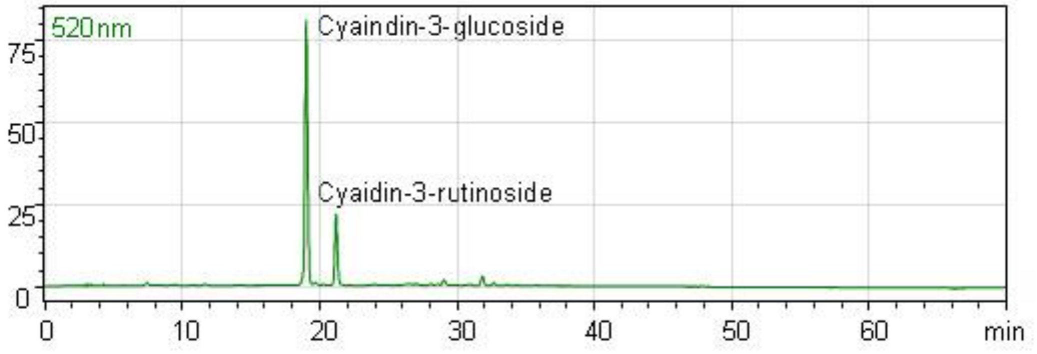

Supplemental Figure S1. Chromatogram of anthocyanin compounds in 'Red Beauty' plum fruit.

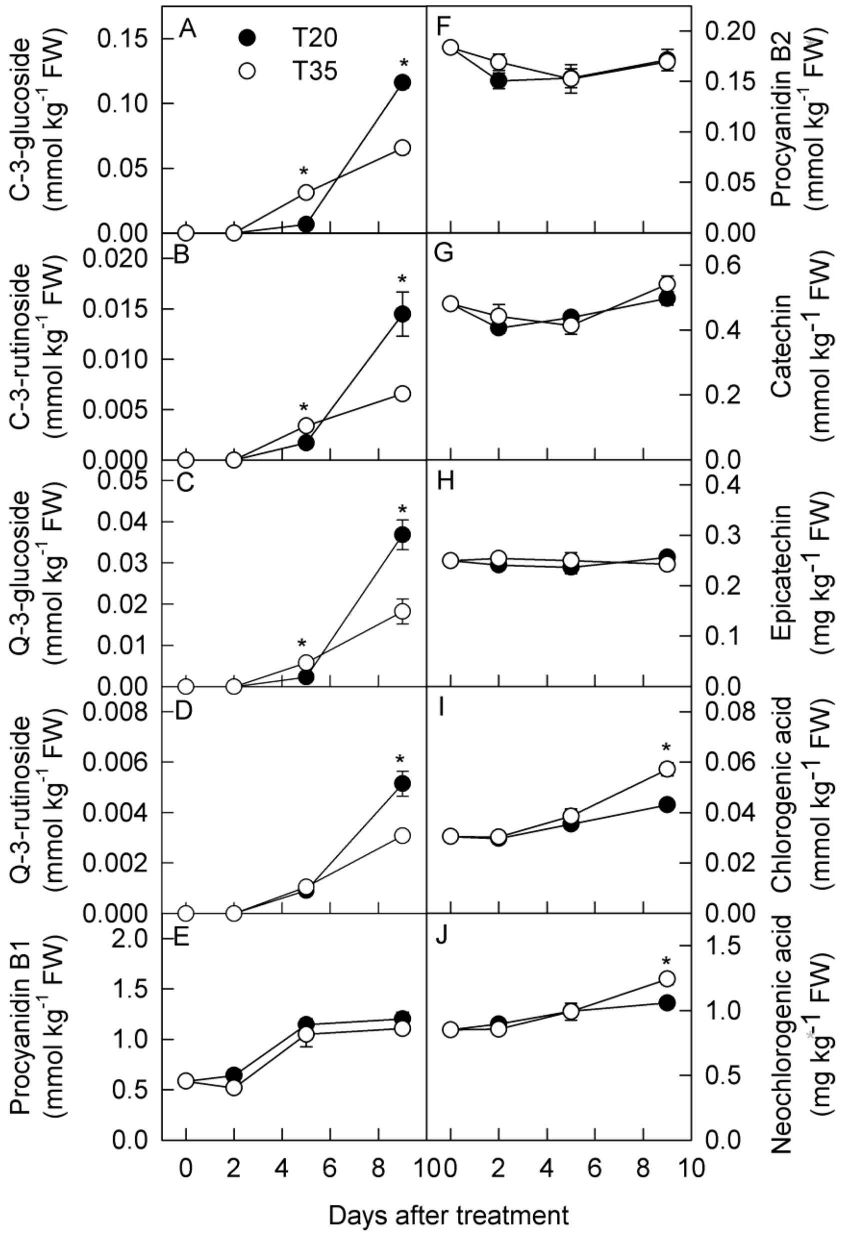

Supplemental Figure S2. The concentrations of phenolic compounds in the fleshes of plum fruits treated at 20 ℃ and 35 ℃ for different times in the dark. Each data point represents the mean ± SE (n = 5). The asterisk indicates a significant difference between two temperature treatments at *P*<0.05 using the *t*-test. C-3-glucoside, cyanidin-3-glucoside; C-3-rutinoside, cyanidin-3-rutinoside; Q-3-glucoside, quercetin-3-glucoside; Q-3-rutinoside, quercetin-3-rutinoside.

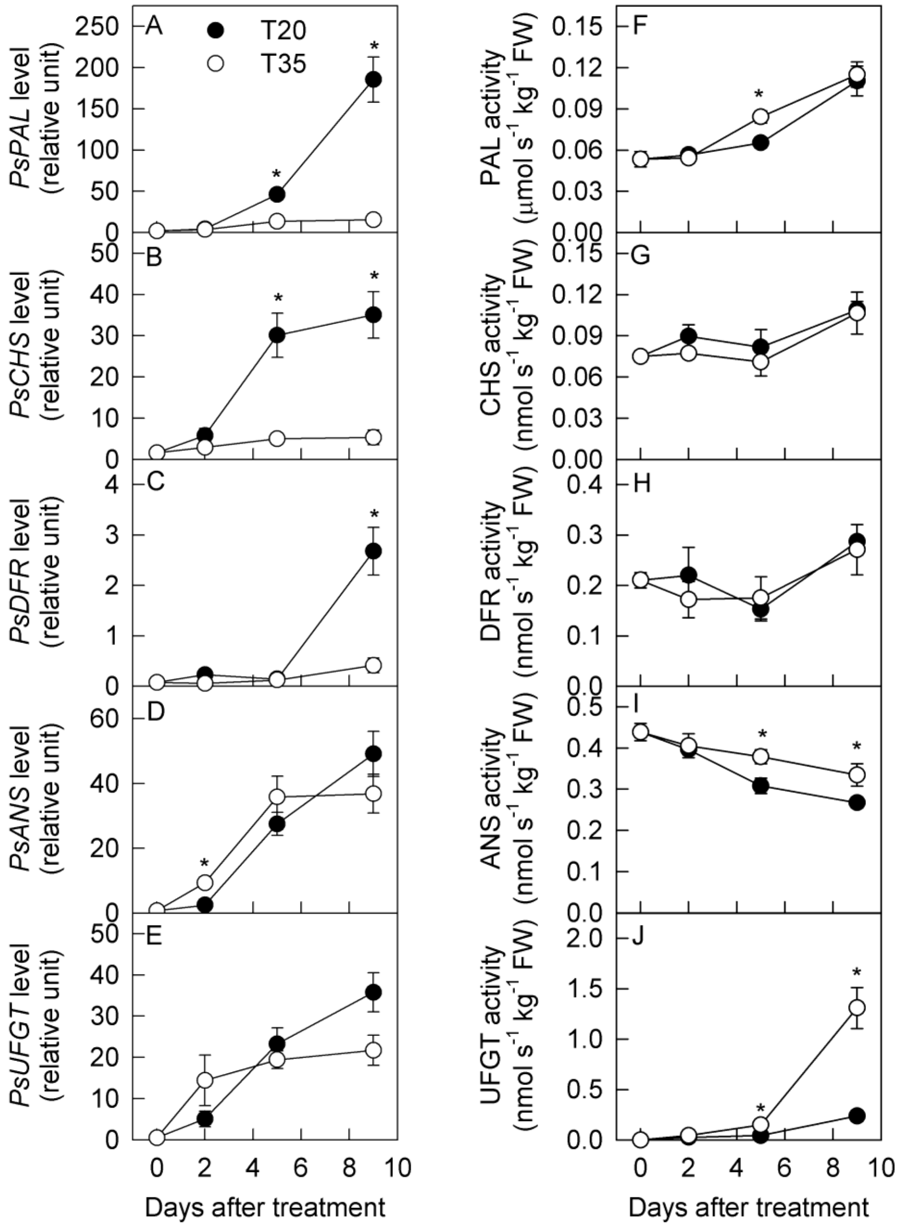

Supplemental Figure S3. The transcription levels of key genes and activities of the corresponding encoded enzymes involved in anthocyanin synthesis in plum fruit fleshes treated at 20 ℃ and 35 ℃ for different times in the dark. Panel A-E, mRNA level; Panel F-J, enzyme activity at 20 ℃. Each data point represents the mean ± SE (n = 5). The asterisk indicates a significant difference between two temperature treatments at $P<0.05$ using the *t*-test. PAL, phenylalanine ammonia-lyase; CHS, chalcone synthase; DFR,

dihydroflavonol reductase; ANS, anthocyanidin synthase; UFGT, UDP glucose:flavonoid 3-O-glucosyltransferase.

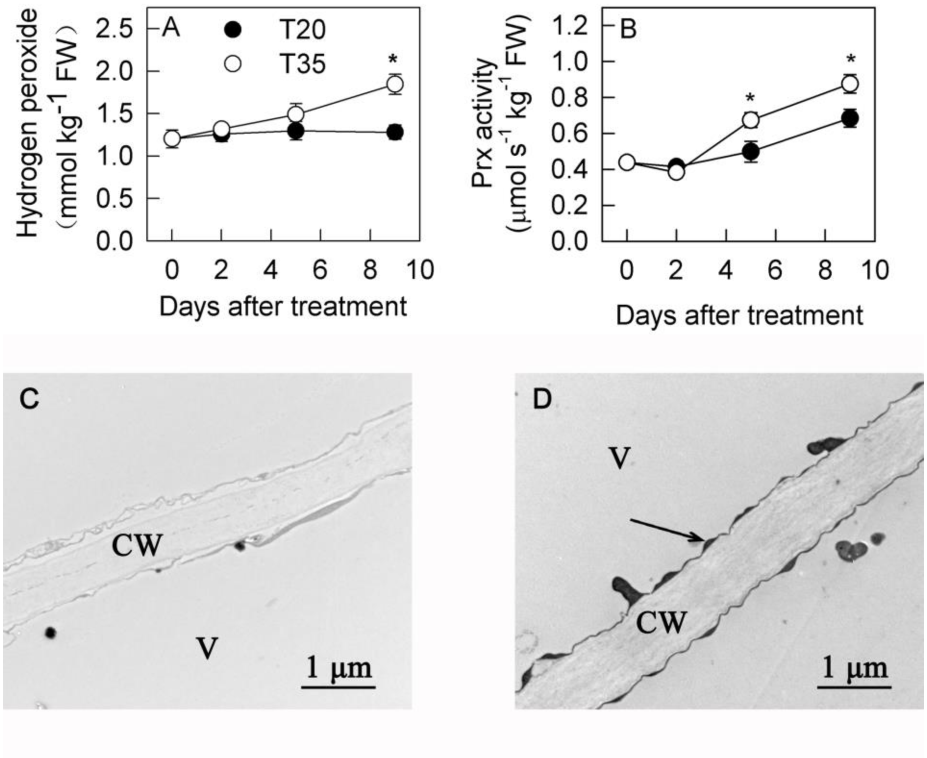

Supplemental Figure S4. Hydrogen peroxide concentration and Class III peroxidase (Prx) activity in the flesh of plum fruits treated at 20 ℃ and 35 ℃ for different durations in the dark. Panel A, hydrogen peroxide concentration in the fleshes during treatments; Panel B, Prx activity in fruit fleshes assayed at 20 ℃; Panel C and D, cytochemical localization of hydrogen peroxide and Prx activity at 20 ℃ and 35 ℃, respectively. Sections were incubated with $CeCl_3$, and the electron-dense deposits represent of both Prx activity and produced hydrogen peroxide in the inner part of the tonoplast (marked with arrows). The vacuolar region is marked with “V”. Bar, 1 μm. Each data point represents the mean ± SE (n = 5) in Panel A and B. The asterisk indicates a significant difference between two temperature treatments at *P*<0.05 using the *t*-test.

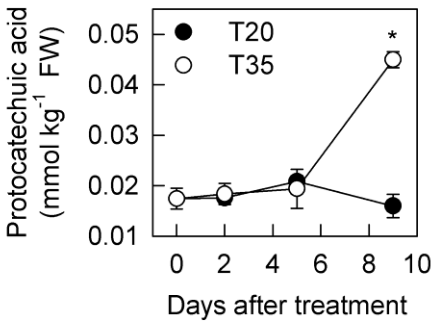

Supplemental Figure S5. Concentrations of protocatechuic acid in the fleshes of plum fruits treated at 20 ℃ and 35 ℃ for different durations in the dark. Each data point represents the mean ± SE (n = 5). The asterisk indicates a significant difference between two temperature treatments at P<0.05 using the *t*-test.

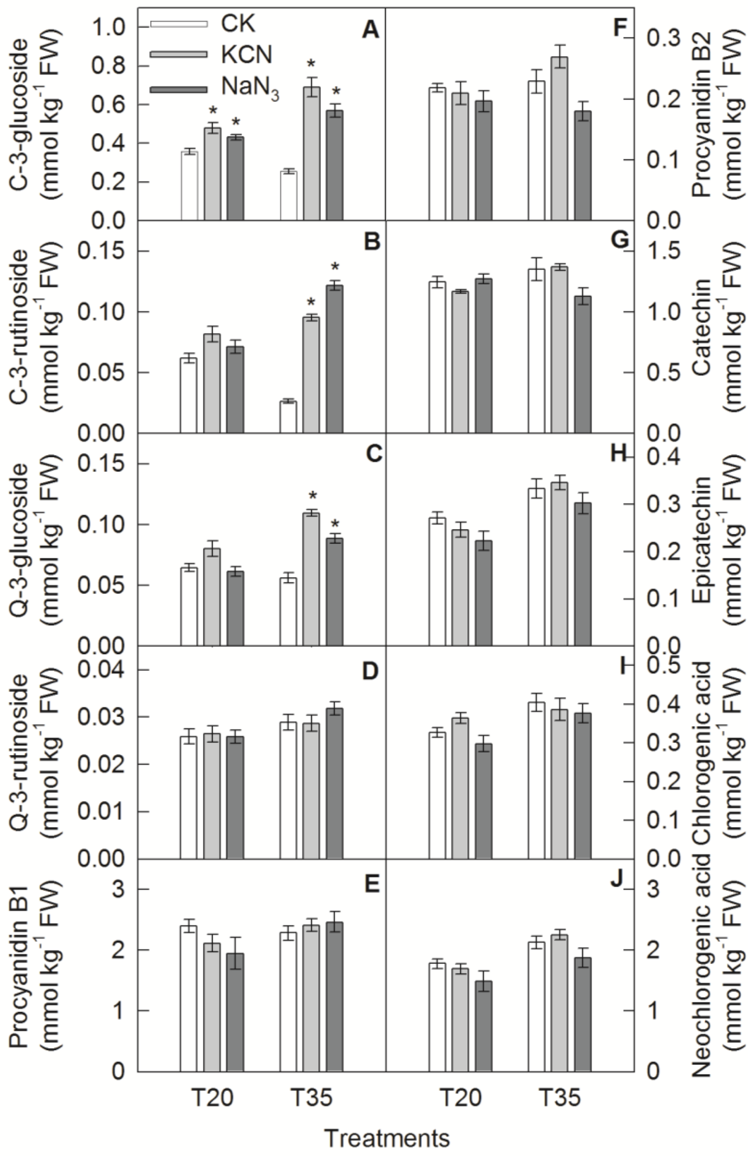

Supplemental Figure S6. Concentrations of phenolic compounds in the peels of plum fruits exposed to different treatments in the dark at 20 ℃ and 35 ℃. Each data point represents the mean ± SE. The asterisk indicates a significant difference between untreated and treated fruits at each temperature at $P<0.05$ using the *t*-test.

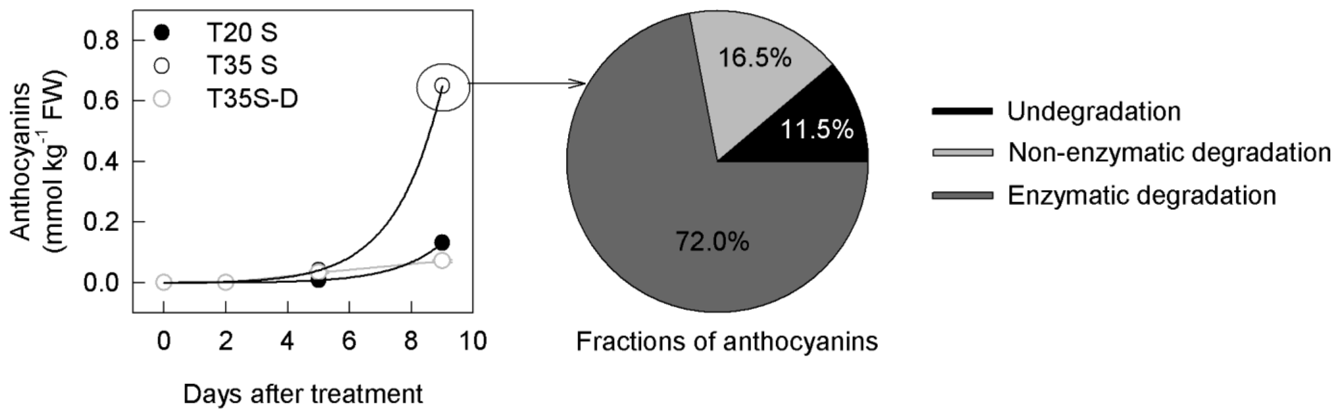

Supplemental Figure S7. The concentrations of grossly synthesized anthocyanin in the fleshes of plum fruits treated at 20 ℃ and 35 ℃ for different durations in the dark (A) and the fractions of anthocyanins in fruit flesh at 35 ℃ on day 9 (B).

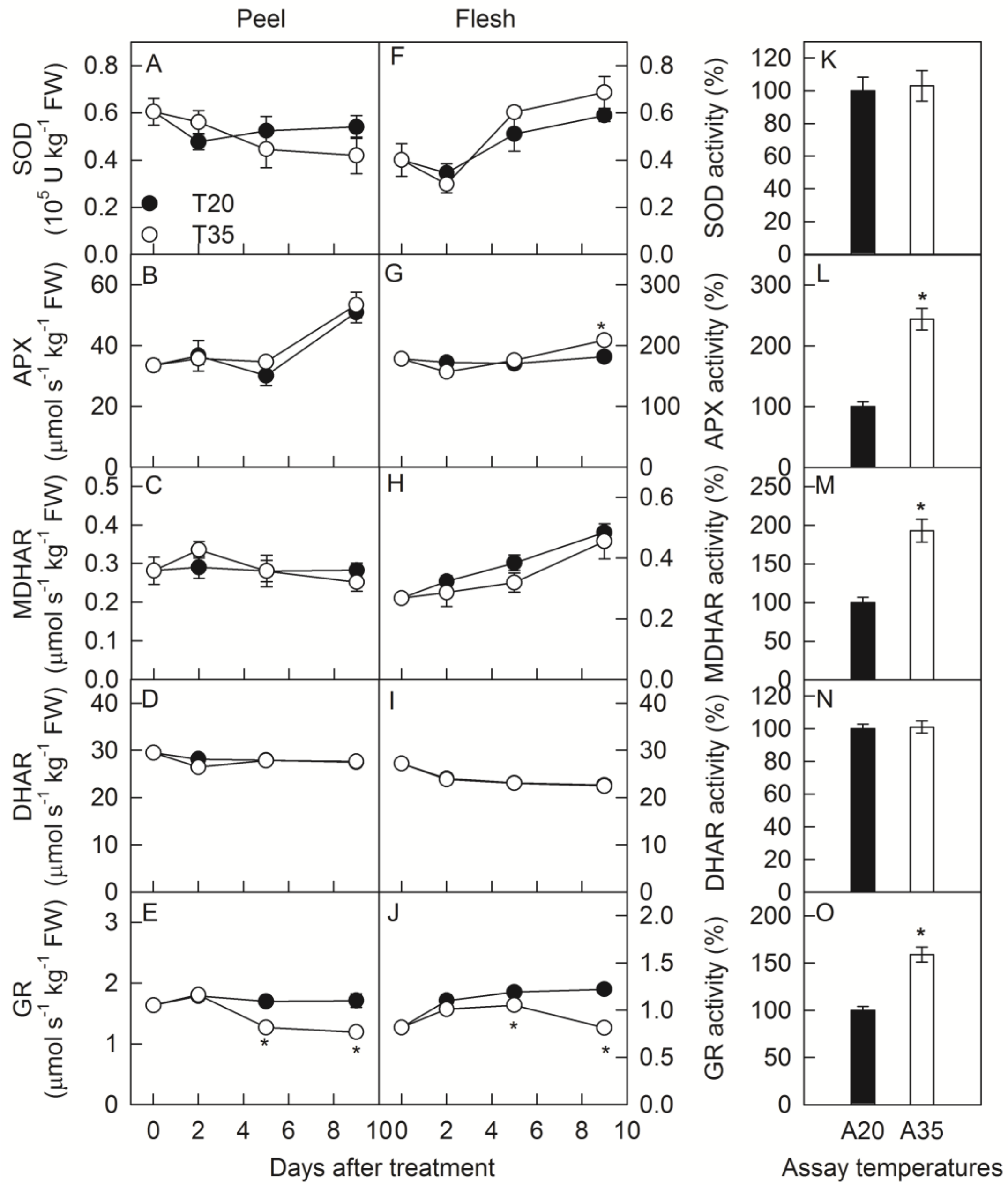

Supplemental Figure S8. The activities of superoxide dismutase (SOD), ascorbate peroxidase (APX), monodehydroascorbate dehydrogenase (MDHAR), dehydroascorbate dehydrogenase (DHAR), and glutathione reductase (GR) in the peels or fleshes of plum fruits treated at 20 ℃ and 35 ℃ for different times in the dark. Panel A-J, enzyme activity at 20 ℃; Panel K-O, enzyme activity in fruit treated at 35 ℃ for 9 days and assayed at 20 ℃ and 35 ℃. Each data point represents the mean ± SE (n = 5, A-J; n = 3, K-O). The

asterisk indicates a significant difference between two temperature treatments at $P<0.05$ using the t-test.

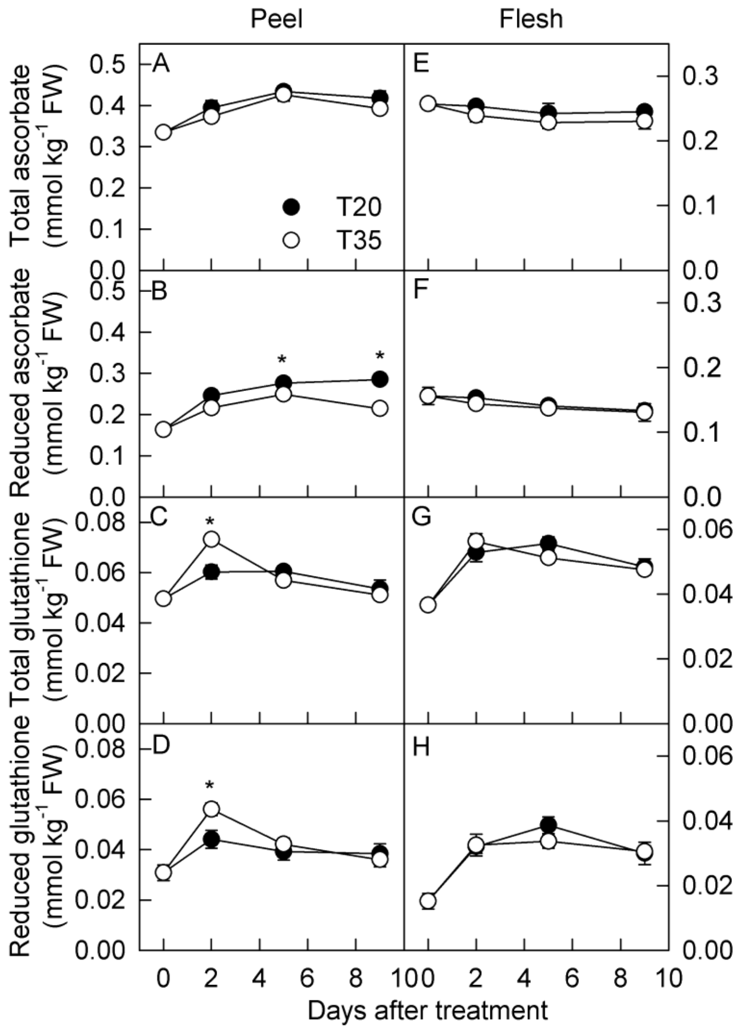

Supplemental Figure S9. The concentrations of total ascorbate, reduced ascorbate, total glutathione, and reduced glutathione in the peels and fleshes of plum fruits treated at 20 ℃ and 35 ℃ for different times in the dark. Each data point represents the mean ± SE (n = 5). The asterisk indicates a significant difference between two temperature treatments at *P*<0.05 using the *t*-test.

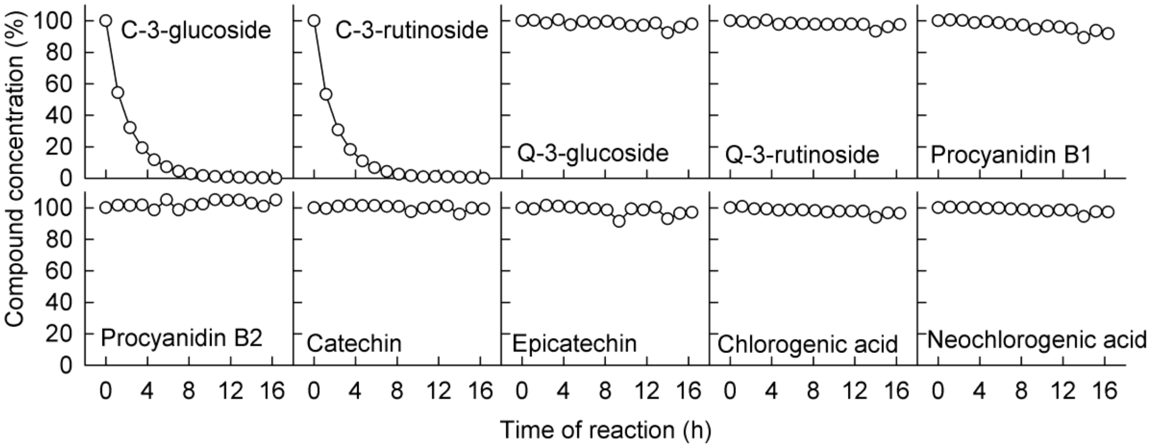

Supplemental Figure S10. Changes in the concentrations of different phenolic compounds in the extract of plum fruit peel reacted with hydrogen peroxide.

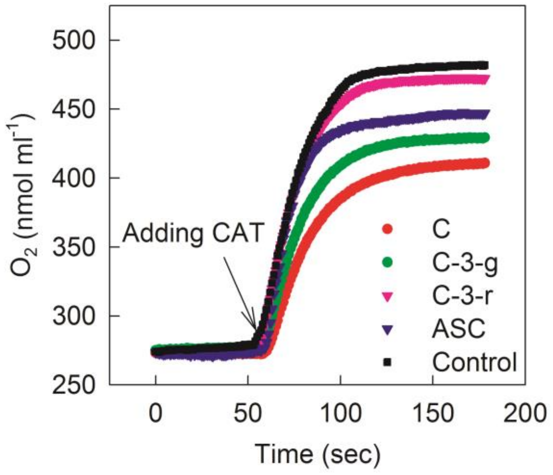

Supplemental Figure S11. The antioxidant capacities of reduced ascorbate (ASC), cyanidin-3-glucoside (C-3-g), cyanidin-3-rutinoside (C-3-r), and cyanidin (C) to scavenge hydrogen peroxide. The O_2 evolution represents the residual hydrogen peroxide after reacting with different compounds with same concentrations. Control, water.

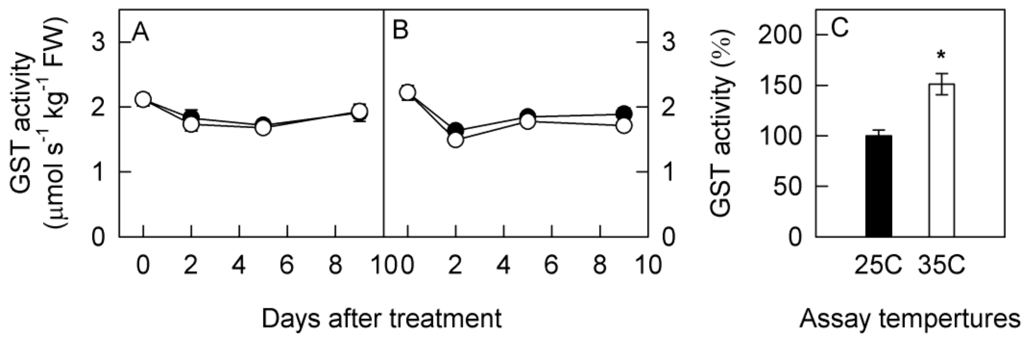

Supplemental Figure 12. The activity of glutathione S-transferase (GST) at 20 ℃ in the peels (A) or fleshes (B) of plum fruits treated at 20 ℃ and 35 ℃ for different times in the dark. Panel C, enzyme activity in fruit treated at 35 ℃ for 9 days and assayed at 20 ℃ and 35 ℃. The asterisk indicates a significant difference between two temperature treatments at P<0.05 using the t-test.
